# Illustrating Key Components to Co-Creation Through Preventive Care mHealth Messaging with Underserved Communities and Expert Partners

**DOI:** 10.1007/s10916-025-02310-z

**Published:** 2025-12-12

**Authors:** Nicole A. Stadnick, Carrie Geremia, Kelli L. Cain, William Oswald, Paul Watson, Marina Ibarra, Men Nguyen, Zainab Altemimi, Noora Hammi, Marlene Bautista, Marwah Alrefaee, Thanh Mai Chu, Nicole M. Wagner, Santosh Vijaykumar, Sean T. O’Leary, Edgar A. Diaz, Jeannette Aldous, Borsika A. Rabin

**Affiliations:** 1 Department of Psychiatry, University of California San Diego, La Jolla, San Diego, CA USA; 2 Altman Clinical and Translational Research Institute Dissemination and Implementation Science Center, University of California San Diego, La Jolla, CA USA; 3 Child and Adolescent Services Research Center, San Diego, CA USA; 4 Department of Medicine, University of California San Diego, La Jolla, San Diego, CA USA; 5 Herbert Wertheim School of Public Health and Human Longevity Science, University of California San Diego, La Jolla, San Diego, CA USA; 6 The Global Action Research Center, San Diego, CA USA; 7 San Ysidro Health, San Diego, CA USA; 8 University of Colorado School of Medicine, Aurora, CO USA; 9 School of Psychology, Northumbria University, Newcastle, England, UK

**Keywords:** Co-creation, MHealth, Community engagement, Underserved communities

## Abstract

Meaningful community engagement is an essential component of impactful public health and implementation research. Multiple community engagement methods have been defined, including co-creation. Co-creation involves an iterative process that advances from identifying opportunities for value creation and solutions, to defining partner priorities, to evaluating co-created outcomes. This study reports our methods to co-create culturally and linguistically meaningful mHealth messages to promote preventive healthcare engagement for Arabic, Spanish, and Vietnamese – speaking communities. This multi-method study is part of a larger program of research, “Working towards Empowered community-driven Approaches to increase Vaccination and preventive care Engagement” (WEAVE), that aims to co-create and test a preventive healthcare program that includes mHealth and care coordination with medically underserved patients at multiple federally qualified health center (FQHC) locations near the US/Mexico border and surrounding region. A multi-level partner process was used to engage in co-creation across six partner groups (*n* = 27): (1) Community Advisory Boards (CAB), (2) Community Weavers (individuals with lived experience as members of an underserved community who act as cultural brokers between communities, public health systems, and researchers), (3) FQHC Care Coordinators, (4) FQHC Administrators, (5) a FQHC Clinical Expert, and (6) Research Experts in health communication, vaccine behavior research, and/or mHealth. Each of these partner groups was distinctly engaged through structured CAB meetings, weekly research and operations team meetings, topic-specific meetings, and e-review of content. The Research Engagement Survey Tool (REST) was used as a global assessment of partner engagement in the co-creation process. Results are organized by a co-creation framework anchored to identify, analyze, define, and design steps. Across four CAB meetings and engagement activities with the other co-creation partners, 262 mHealth messages (89 Arabic, 85 Spanish, 88 Vietnamese) were refined and approved. A message cadence and delivery mode were finalized. On the REST, the average ratings were over 4.50 (out of 5), indicating strong perceptions of engagement with the co-creation process and members. We successfully engaged six co-creation partner groups to develop and approve the content, cadence, and delivery mode of mHealth preventive care messages. These messages will be embedded in the multicomponent health program that will be tested in a randomized adaptive trial. NCT05841810, registration date: 03/28/2023.

**Supplementary Information:**

The online version contains supplementary material available at 10.1007/s10916-025-02310-z.

## Background

Public health research and practice demands meaningful community engagement to promote effective, equitable, and sustainable programs and service delivery.[1, 2] Community engagement methods may vary in their purpose, goals, resource investment, and level of engagement.[3] The intentional engagement of community members or partners in influencing health services, implementation, and evaluating impact has been referred to as co-design, co-creation, and co-production.[4] These are distinct terms that vary in their focus, engagement approach, and products developed. In this study, we focus on co-creation, conceptualized as the overarching process of engagement that is guided by the principles of co-creating value, focusing on all partners’ experiences, direct interaction between partners, and creating communication platforms that promote consistent dialogue between partners.[5] The co-creation process involves iterative progression through multiple steps, from identification of opportunities for value creation and solutions, to partner priorities, to evaluation of the co-created outcomes. Co-creation involves a high level of active and ongoing collaboration across partners who uniquely contribute their perspectives and expertise to problem-solve, design, implement, and evaluate aspects of the effort. We briefly review the co-creation steps:

1. **Identify** all partners relevant to the effort of interest to clarify opportunities for value creation and solutions to advance the effort of interest.
2. **Analyze** the partner network, identify shared and conflicting values of partners, clarify the processes and options for decision making and obligations among the partners.
3. **Define** and prioritize challenges, next steps, and actions to address the effort of interest.
4. **Design** solutions through collaborative goal setting, actions to achieve the goals, evaluation processes, and equitable allocation of resources.
5. **Realize** the potential effects of the co-created solutions during implementation when partners test the co-created product.
6. **Evaluate** the proposed outcomes, the success of the previous steps, the learnings from the diverse partners, and plans for sustainability.

Growing demand for co-creation in public health comes at a time when there has been rapid rise in mobile health (mHealth) technologies to expand service capacity and reach, promote healthcare engagement, and reduce disparities in access and health outcomes.[6] The use of digital health products, particularly digital mental health products, significantly increased during the COVID-19 pandemic era.[7] Balanced with the benefits and opportunities of mHealth are important considerations for implementation in real world settings, including technology literacy, personalization of interventions, and perceptions of health risk and status.[8] Understanding these considerations can inform appropriate selection of implementation strategies tailored for context and implementation phase [9].

The current study is part of a larger program of NIH-funded research referred to as “Working towards Empowered community-driven Approaches to increase Vaccination and preventive care Engagement” (WEAVE). The WEAVE study aims to increase engagement with age- and gender- based preventive care behaviors including vaccine uptake by co-creating and testing a multicomponent health program that includes mHealth outreach and care coordination for and with underserved communities receiving care at a federally qualified health center (FQHC)[10]. Adult patients who speak Spanish, Vietnamese, or Arabic were prioritized by the partnering FQHC because they had identified need to increase healthcare engagement with these patient groups. The preventive care behaviors were also prioritized by the partnering FQHC (and national public health priorities), and include influenza vaccine, COVID-19 vaccine, breast cancer screening, colorectal cancer screening, and cervical cancer screening.

The purpose of this study is to describe our methods to co-create culturally and linguistically meaningful mHealth messages that promote vaccine uptake and engagement in preventive healthcare and our assessment of engagement in the co-creation process.

## Methods

### Participants and Procedures

An iterative and multi-perspective process was used to engage in co-creation with six partner groups: (1) Community Weavers, (2) Community Advisory Boards, (3) FQHC Care Coordinators, (4) FQHC Administrators, (5) FQHC Clinical Expert, and (6) Research Experts in health communication, vaccine hesitancy, and/or mHealth interventions. Below, we describe the composition of each co-creation partner group and procedures for engaging them in co-creation. Table 1 summarizes the number and roles of the partners who participated in the co-creation process. Table 2 summarizes the co-creation process across partner groups.

**Table 1:** Participants by role in the co-creation process

Partner Group	*N*
CAB members	15
Community Weavers	3
FQHC Care Coordinators	3
FQHC Administrators	2
FQHC Clinical Expert	1
Research Experts	3
Total (All Co-Creation Partners)	27

**Table 2:** Contributions from each co-creation partner on message co-creation

	Method & Frequency of Co-creation	Message Alignment with Health Recommendations	Message Cultural appropriateness	Message Fit with FQHC	Message Language Accuracy	Message Format	Message Cadence	Message Timing/ Frequency
Co-Creation Partner Group		How well does the message align with public health and clinical recommendations?	How well does the message align with cultural preferences and norms within the priority community?	How well does the message fit with the care processes of the FQHC?	How well does the message use accurate language?	How appropriate is the format (text, video, voice message) of the message delivery?	In what order should messages be sent?	What is the best timing (day of week/time of day) and frequency for the delivery of the messages?
Community Weavers	- Meeting with Global ARC supervisor (weekly) - Research team meetings (monthly) - Email (as needed)		X		X	X	X	X
CAB	- 4 virtual meetings		X		X	X	X	X
FQHC Care Coordinators	- Meetings with FQHC supervisor (weekly) - Research team meetings (bi-monthly) - Email (as needed)		X		X	X	X	X
FQHC Administrators	- Research team meetings (weekly) - Additional meetings as needed - Email (as needed)	X		X	X	X	X	X
FQHC Clinical Expert	- Study launch meeting (one time) - Email (one time)	X						
Research Expert - Health Communication	- Study launch meeting (one time) - Topic-specific meeting (two times) - Email (as needed)	X	X			X		
Research Experts - Vaccine Hesitancy and mHealth Intervention Design and Evaluation	- Topic-specific meeting (three times) - Email (as needed)	X	X			X	X	X

1. *Community Weavers*. Community Weavers are individuals with lived experience as members of an underserved community who act as cultural brokers between communities, public health systems, and researchers to co-create community-driven public health solutions.[11, 12] Three Community Weavers were identified, trained, and supervised by the Global Action Research Center (ARC), a non-profit social change organization with expertise conducting participatory action research to address public health and environmental justice needs. The Global ARC is considered a trusted resource for many grassroot organizations in the broader San Diego area. Each Community Weaver represented one of our priority communities, was bilingual in English plus Spanish, Arabic, or Vietnamese, and identified and invited CAB members to join the study CAB based on their community network. CAB sessions were led by Community Weavers, and the meetings were conducted in the CAB members’ preferred language (i.e., Spanish, Arabic, or Vietnamese). Community Weavers were engaged in the co-creation process by participating in the development and translation of mHealth-related CAB meeting materials, leading CAB meetings, reviewing mHealth content for alignment of cultural preferences and norms within the priority community, and translating or reviewing translations of mHealth content. Community Weavers participated in weekly meetings with the Global ARC, joined research team meetings approximately once per month, and responded to email requests to review and translate mHealth messages. The initial mHealth messaging concept came from collaboration between the research team and the Global ARC. While the research team introduced the concept of mHealth outreach as a potentially scalable communication strategy, the Global ARC and Community Weavers ensured that the messages were culturally aligned with community norms and values. The Community Weavers, drawing on their lived experiences and ongoing dialogue with CAB members, guided early discussions on trust and accessibility of mobile messaging within their communities. During these discussions, partners acknowledged that trust in digital communication varied across language groups, particularly among older adults and recent immigrants. For this reason, alternative modes (e.g., voice recordings, printed reminders, or phone calls) were retained as supplementary options discussed with the CAB. Ultimately, mHealth was prioritized given its widespread use across the three communities and its potential alignment with FQHC outreach infrastructure.
2. *Community Advisory Boards (CAB).* Three CAB were established through recruitment of community members by the Community Weavers (i.e., individuals with lived experience as members of an underserved community serving as cultural brokers between communities, public health systems, and researchers to co-create community-driven, culturally sensitive public health solutions) who identified and invited CAB members to join their CAB based on their community network. Each CAB included five adult CAB members who identified as a leader, member, and/or advocate for one of three communities: (1) Spanish-speaking, (2) Arabic-speaking, and (3) Vietnamese-speaking. There were 15 unique CAB members across three CAB. The emphasis on specific language groups to define the CABs aligns with the healthcare engagement s and communications needs of the partnering’s FQHC patient population. Specifically, the FQHC prioritized healthcare engagement gaps for their Spanish-speaking, Arabic-speaking, and Vietnamese-speaking patients. Each CAB met four times between August 2023 and June 2024. Table 3 describes the topics discussed in each CAB session.

**Table 3:** CAB session topics

Meeting	Topics	Goals
Meeting #1	• Welcome and overview of the WEAVE study • Overview including ground rules and roles for the CAB • Consent for video recording • Overview of community engagement – why, how, what • Discuss meeting procedures and processes • Closing and next steps	Establish the CAB rules of engagement and orient the CAB to the WEAVE study and future activities.
Meeting #2	• Welcome • Review priority preventive care behaviors and recommendations • Review messaging content strategies • Review preferred messaging format options • Closing and next steps	Receive feedback on general message content and strategies such as reminders, providing encouragement and providing information for preventive care behaviors; feedback about the community’s preferred forms of communication such as text messages with words or voice recordings, images, email, and print; and feedback about the community’s preferred timing and cadence of mHealth messages.
Meeting #3	• Welcome • Show sample message content for each behavior and ask 3 questions: o Are these messages understandable to your community? o Are these messages meaningful to your community? o Do you notice anything offensive or inappropriate? • Questions about communication o What is the most effective way to communicate with your community? o How often texts would members of your community be open to receiving texts? o What is the maximum number of texts per week we should send to your community? • Closing and next steps	Gather reflections on a three-tier messaging system and drafts of mHealth messages developed to address specific missing health behaviors.
Meeting #4	• Welcome • Show final messages for each missing behavior and ask 3 questions: o Are these messages understandable to your community? o Are these messages meaningful to your community? o Do you notice anything offensive or inappropriate? • Ask CAB members to order the set of messages considering the priorities and preferences of their community • Closing and next steps	Receive feedback on behavior-specific message content as well as the order in which messages for each specific missing health behavior will be delivered.

Briefly, the first session focused on introductions, establishing CAB rules of engagement, orientation to the WEAVE study, and previewing the focus of the upcoming sessions that focused on content, delivery, and mode of mHealth message outreach. The second session focused on general messaging content (reminders, encouragement, providing information), the preferred form of communication (e.g., text message with words or voice recordings, images, email, print), and timing and cadence of mHealth messages. The third session focused on gathering reflections on a three-tier messaging system, in which the content of messages would increase in urgency, and drafts of mHealth messages developed to address specific missing health behaviors. CAB members were asked whether the messages were understandable to their community, meaningful to their community, and if they noticed anything offensive or inappropriate in the messages. The fourth session focused on behavior-specific message content and flow of the message delivery. CAB members were asked for final reflections on the message content relative to the priorities and preferences of their communities and for preferences for the order in which messages are delivered for each behavior.

Each CAB session was led by a Community Weaver and conducted in the primary non-English language of the CAB (Spanish, Arabic, or Vietnamese). There was an English interpreter who provided live interpretation and translation for the English-speaking Global ARC and research team members. At each CAB session, a Care Coordinator from the partnering FQHC, fluent in the relevant language and familiar with the community, was present, and there were 2–3 members of the research team who provided research study clarifications and actively listened to the discussion. The research team (Principal Investigators and core research staff) participated as observers and aids to structuring co-creation content. All sessions were audio-recorded (with permission) and transcribed in the CAB primary language and English. Each CAB session lasted 90 min to 2 h and was conducted virtually using the Zoom platform. Each CAB member received a $100 stipend per meeting for their participation.

3. *FQHC Care Coordinators*. Three FQHC Care Coordinators who are also members of the three study communities and bilingual in English and Spanish, Arabic, or Vietnamese were hired and supervised by the partnering FQHC. Care Coordinators were engaged in the co-creation process by attending CAB meetings for their community, reviewing mHealth messages for alignment with cultural preferences and norms within the priority community, and translating or reviewing translations of mHealth content. Care Coordinators met weekly with FQHC Administrators, joined research team meetings twice monthly, and responded to email requests to review and translate mHealth messages.
4. *FQHC Administrators*. Two FQHC Administrators (Health Support Services Manager and Director of Research and Health Promotion) were engaged in the co-creation process by contributing to the development and refinement of mHealth message content, participant surveys, consent forms, and recruitment materials. The Health Support Services Manager joined all CAB meetings, participated in weekly research team meetings, and supervised Care Coordinator activities. The Director of Research and Health Promotion joined research team meetings monthly. Both Administrators responded to regular email requests about the larger research study and FQHC Care Coordinator activities and participated in topic-specific Zoom meetings with the research team to review mHealth messages related to the fit with current care processes at the FQHC.
5. *FQHC Clinical Expert.* The FQHC’s Clinical Director of Infectious Disease engaged in the co-creation process by participating in the study launch meeting, contributing to discussions about the content of mHealth messaging, and responding to email requests to review mHealth messages developed with the CABs for alignment with public health and FQHC clinical recommendations.
6. *Research Experts*. Three Research Experts with expertise in health communication, vaccine hesitancy, and mHealth intervention design and evaluation engaged in the co-creation process by participating in the study launch meeting, contributing to discussions about the content of mHealth messaging, and participating in small group meetings with the research team targeted to their specific research expertise. The Research Experts also responded to email requests to review mHealth content developed with the CABs to advise on the format, content, cadence, timing, and frequency of messages. Their input drew upon established behavior change and persuasion frameworks, including the Health Belief Model [13] and Framing Theory [14], to ensure that message content highlighted perceived susceptibilities and benefits and also framed information in ways that emphasized positive, actionable health outcomes and avoided losses. This guidance ensured that theoretical constructs were integrated with community priorities and feedback, resulting in an mHealth intervention that was both theoretically grounded and culturally appropriate.

### Measures

We assessed the quality and frequency of engagement with the co-creation partners using the Research Engagement Survey Tool (REST*).*[15] This 9-item measure assesses the quality and frequency of 8 partner engagement principles: (1) Focus on community perspectives and determinants of health, (2) Partner input is vital, (3) Partnership sustainability to meet goals and objectives, (4) Foster co-learning, capacity building, and co-benefit for all partners, (5) Build on strengths and resources within the community or patient population, (6) Facilitate collaborative, equitable partnerships,7) Involve all partners in the dissemination process, 8) Build and maintain trust in the partnership. Each item is measured on a 5-point Likert-scale with 0 = poor and 5 = excellent. CAB members completed this measure after each CAB session. To describe perceived engagement, an average score across all CAB sessions was used for the CAB members. The additional co-creation partner groups (Community Weavers, FQHC Care Coordinators, FQHC Administrators, FQHC Clinical Expert, and Research Experts) completed the REST at the end of the first phase of mHealth message development with instructions to rate engagement with the research team in the process of the mHealth co-creation since the start of the study.

## Results

The results are organized using the co-creation process described by Vargas and colleagues [5]. Across each step, the research team facilitated the convening of our co-creation partners and rigorously assessed their interactions and synthesize their feedback. For this study, we describe our co-creation process and key lessons learned across the first four steps: identify, analyze, define, and design.

### Identify: Meeting #1

In meeting #1, CAB members, meeting facilitators, FQHC administrators, and FQHC Care Coordinators were introduced. CAB rules and procedures were established, and a broad-level overview of the study was presented along with the expectations and activities for the CABs. Evaluation activities were introduced, and consent was obtained for evaluation activities from CAB members. The overall goal of this meeting was to identify opportunities for value creation (e.g., to co-create content for mobile health outreach to ensure culturally competent messaging; to co-create and support evaluation of care coordination services that promote preventive care engagement) and partner priorities (e.g., the primary motivation for each CAB member to participate in their CAB).

### Analyze: Meeting #2

**Fig. 1:**
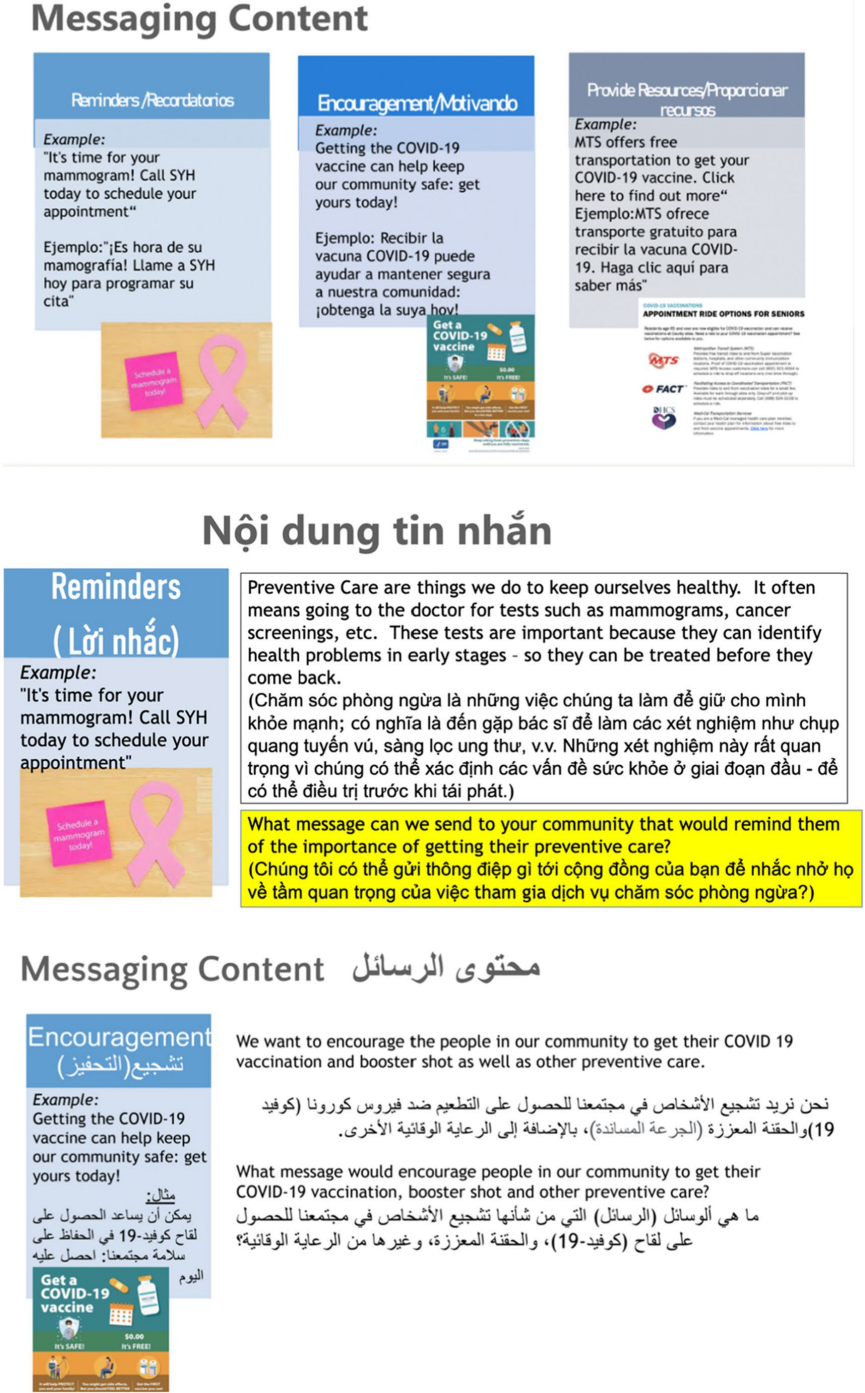
Types of mHealth messages presented to the CAB

Three broad content themes of mHealth messages: reminders, encouragement, and providing resources/information were developed with input from health communications Research Experts prior to Meeting #2. The sequencing of message types was intentionally structured to move from general awareness and motivation (encouragement) toward practical support (information) and specific action prompts (reminders). This scaffolding was designed to grow patients’ readiness, confidence, and engagement with preventive care behaviors. In meeting #2, CAB members were introduced to these three content themes and examples of each messaging type. Figure 1 shows an example. The discussion also covered the preferred modalities of delivery: text, voice messages, images, email, and print. The results of these discussions guided the next step in bidirectional message creation, informing refinement and edits.

### Define: Meeting #3

**Fig. 2:**
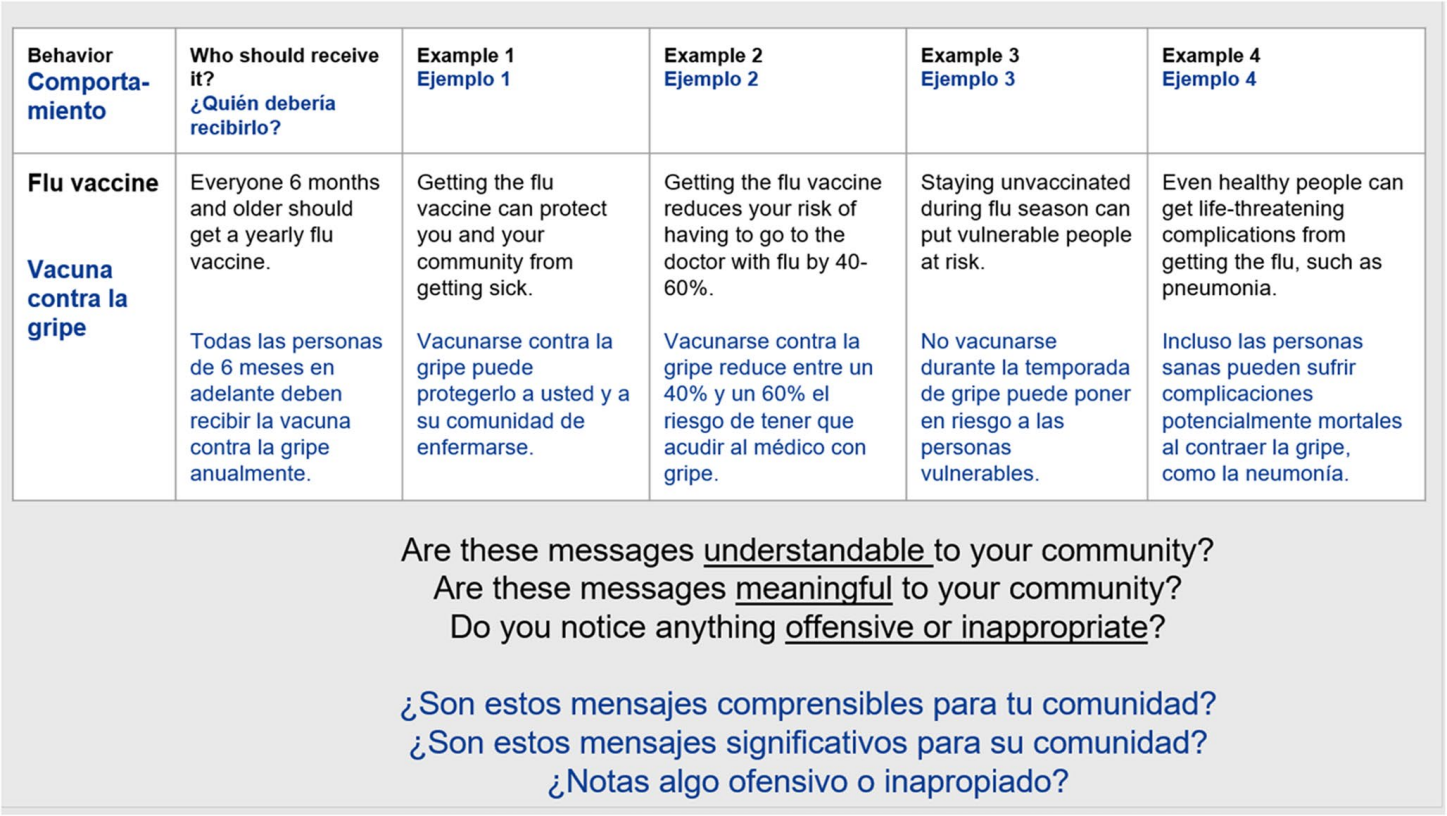
Example mHealth message content presented to the CAB

In the third CAB meeting, participants were shown specific examples of mHealth messages targeting a specific health behavior. They were then actively encouraged to reflect on and discuss the ease of understanding, meaningfulness, and appropriateness of the messages within their community context (see Fig. 2). Interactive discussions were encouraged, with CAB members actively engaged in suggesting modifications to the message wording, content, and delivery strategy. In total, 24 messages were reviewed for cultural appropriateness and translated into the study languages by the FQHC Care Coordinators with translations being reviewed and approved by the Community Weavers. These messages were then reviewed and approved by each CAB in their preferred language. The research team then incorporated this feedback into subsequent iterations of the mHealth messages according to the community’s preferences and needs. CAB members were then invited to reflect on their community’s preferences for the timing and cadence of mHealth text messages. Finally, these messages were reviewed by FQHC Administrators, FQHC Clinical Expert, and Research Experts for alignment with public health and clinical recommendations, fit with the care processes of the FQHC, and cultural appropriateness. After review, two modifications were suggested.

### Design: Meeting #4

**Fig. 3:**
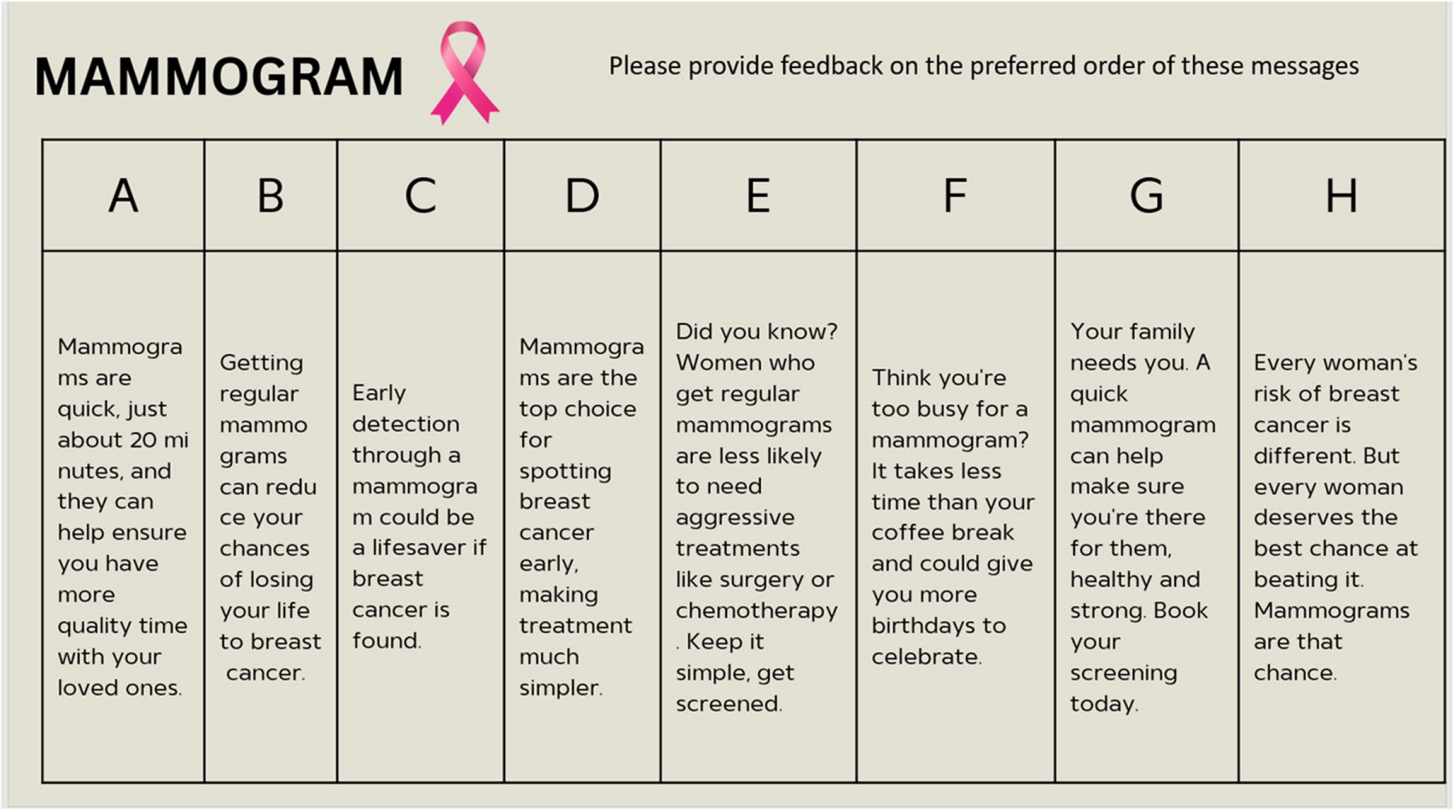
Example mHealth messages presented to the CAB for feedback on delivery order

In the fourth CAB meeting, participants were presented with the final behavior-specific mHealth messages that had been iteratively modified based on feedback from CAB members and co-creation partners. CAB members were asked to give final approval of message wording and content, including translations, and to suggest any further wording changes. Each CAB reviewed 90 messages in total in their preferred language. The Spanish-speaking CAB suggested modifications to 3 messages, to combine 2 messages into 1, and approved 85 messages. The Arabic-speaking CAB suggested modifications to 1 message and approved 89 messages. The Vietnamese-speaking CAB suggested combining 2 messages into 1 and approved 88. The research team then asked for feedback about the preferred order of message delivery within each behavior (see Fig. 3). The order of message delivery was established through the voting by each CAB member and resulted in a unique cadence for each CAB that is shown in Additional file 1.

## Research Engagement Survey Tool (REST)

Participants rated the quality and quantity of engagement as overwhelmingly positive for all 8 partner engagement principles using the REST tool. The mean scores for ‘quality’ of engagement ranged from 4.50 (out of 5) for focus on community perspectives and determinants of health to 4.84 (out of 5) for build and maintain trust in the partnership. The mean scores for ‘quantity’ of engagement ranged from 4.54 (out of 5) for partner input is vital to 4.95 (out of 5) for build and maintain trust in the partnership. There was some variation in scores among co-creation partner groups, but all engagement principles were rated as very good or excellent and often or always for quality and frequency, respectively (see Table 4).

**Table 4:** Mean quality and quantity scores for REST partner engagement principles for co-creation partners

Group	Quality (how well), M (SD) Quantity (how often), M (SD)						
	Focus on community perspectives and determinants of health	Partner input is vital	Partnership sustainability to meet goals and objectives	Foster co-learning, capacity building, and co-benefit for all partners	Build on strengths and resources within the community or patient population	Facilitate collaborative, equitable partnerships	Build and maintain trust in the partnership
All groups (*n* = 24)	4.50 (0.69) 4.60 (0.66)	4.51 (0.76) 4.54 (0.67)	4.62 (0.83) 4.62 (0.87)	4.61 (0.69) 4.73 (0.46)	4.52 (0.67) 4.63 (0.47)	4.66 (0.56) 4.76 (0.42)	4.84 (0.56) 4.95 (0.37)
CAB (*n* = 15)	4.33 (0.72) 4.52 (0.71)	4.22 (0.80) 4.52 (0.71)	4.25 (0.87) 4.44 (0.92)	4.39 (0.73) 4.64 (0.49)	4.41 (0.70) 4.64 (0.49)	4.57 (0.60) 4.70 (0.46)	4.56 (0.58) 4.74 (0.41)
Community Weavers (*n* = 2)	4.50 (0.71) 4.50 (0.71)	5.00 (0.0) 4.50 (0.71)	4.50 (0.71) 4.00 (1.41)	5.00 (0.0) 5.00 (0.0)	4.50 (0.71) 4.50 (0.71)	4.75 (0.35) 4.75 (0.35)	5.00 (0.0) 5.00 (0.0)
FQHC Care Coordinators (*n* = 3)	4.67 (0.58) 5.00 (0.0)	4.33 (0.58) 4.67 (0.58)	4.33 (0.58) 4.67 (0.58)	4.67 (0.58) 5.00 (0.58)	4.67 (0.58) 5.00 (0.0)	4.50 (0.50) 4.83 (0.29)	4.67 (0.58) 5.00 (0.0)
FQHC Administrators (*n* = 1)	4.00 (0.0) 4.00 (0.0)	4.00 (0.0) 4.00 (0.0)	5.00 (0.0) 5.00 (0.0)	4.00 (0.0) 4.00 (0.0)	4.00 (0.0) 4.00 (0.0)	4.50 (0.0) 4.50 (0.0)	5.00 (0.0) 5.00 (0.0)
Research Experts (*n* = 3)	5.00 (0.0) 5.00 (0.0)	5.00 (0.0) 5.00 (0.0)	5.00 (0.0) 5.00 (0.0)	5.00 (0.0) 5.00 (0.0)	5.00 (0.0) 5.00 (0.0)	5.00 (0.0) 5.00 (0.0)	5.00 (0.0) 5.00 (0.0)

*FQHC Clinician did not complete the survey

**Fig. 4:**
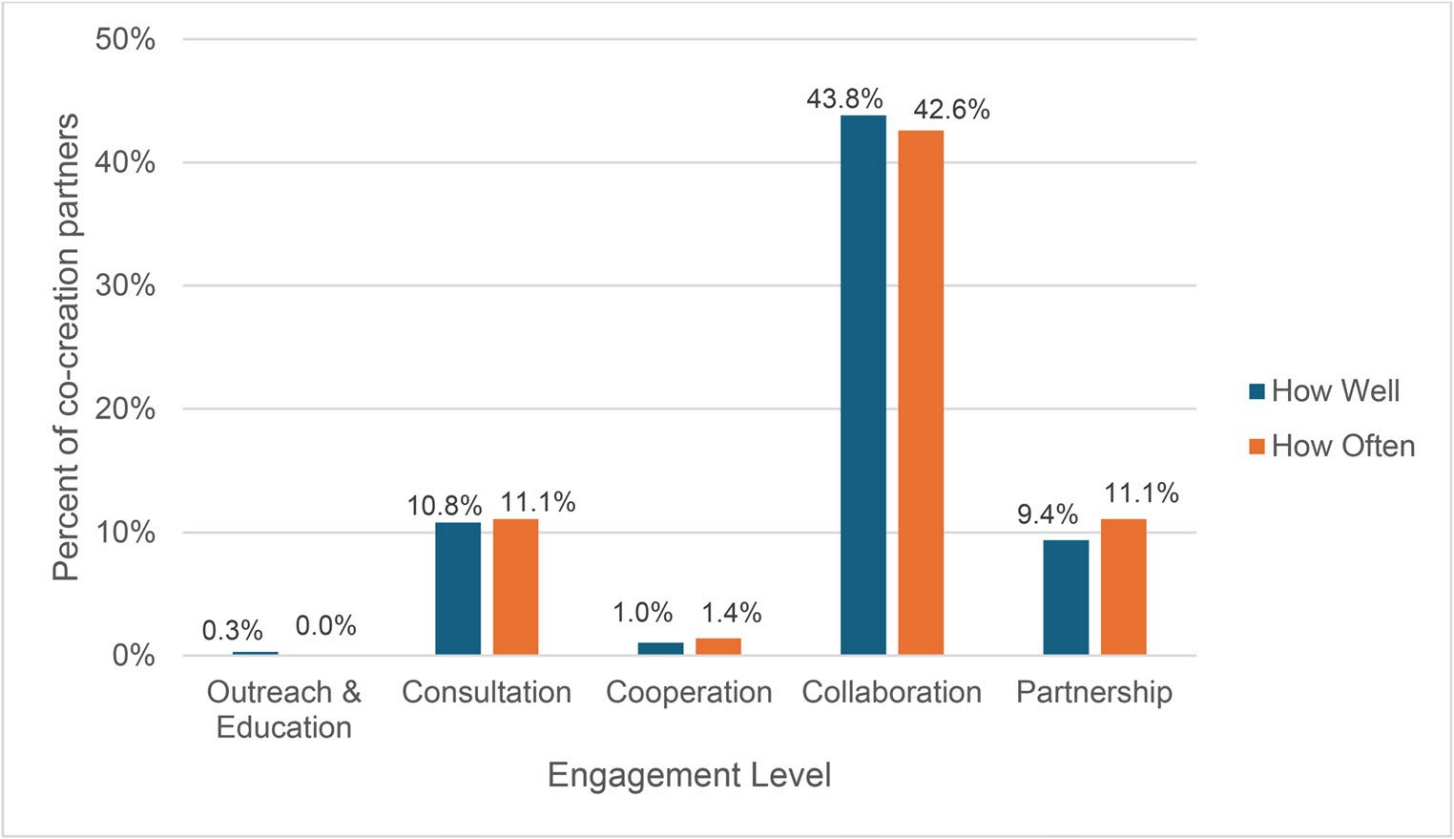
REST levels of engagement (quality and quantity) reported by co-creation partners

REST item responses were then classified into five categories of engagement: Outreach & Education, Consultation, Cooperation, Collaboration, and Partnership according to Goodman et al., 2020. These levels of partner engagement are considered be on a continuum with Outreach & Education & Consultation as earlier forms of engagement and Partnership as later, richer forms of engagement. Scores for each level of engagement were averaged across all participants and shown in Fig. 4. Most co-creation partners classified this project as a collaboration (43.8% and 42.6% for quality and quantity, respectively) or partnership (9.4% and 11.1%) with about 10% classifying it as a consultation (10.8% and 11.1%). These results were consistent across co-creation partners.

Based on our co-creation steps and REST survey results, we synthesized key lessons learned.

1. Inclusive and Iterative Partner Engagement
  Our co-creation approach engaged multiple partner types across multiple timepoints, from planning through implementation.
2. Community-Led Co-Creation Structure
  The Global ARC, a trusted community organization, led hiring and supervision of Community Weavers.
  Community Weavers then identified and led each CAB, facilitating authentic community leadership.
  The researchers primarily acted as facilitators, observers, and expert consultants outside of CAB sessions, creating space for community-driven processes.
3. Thoughtful Partner Selection and Attention to Power Balance
  Partner selection was thoughtful and intentional to reflect who would be involved in mHealth implementation: community members, FQHC patients, FQHC providers, and FQHC administrators.
  The research team recognized and aimed to mitigate potential power imbalances by elevating the leadership role of the CABs to the Global ARC and the Community Weavers.
4. Cultural and Linguistic-Centeredness and Responsiveness
  CAB sessions were conducted in members’ preferred languages and led by Community Weavers who were members of their CAB members’ local communities.
  Co-creation of mHealth messages with each CAB centered on the cultural values, preferences, and norms of each group.
5. Iterative, Scaffolded Learning and Feedback
  Our co-creation approach employed bi-directional and iterative learning methods with feedback progressing from broad concerns about mHealth content to targeted, specific refinements on content, delivery modes, language accuracy, and cultural relevance.
6. Communication and Coordination Considerations
  Communication frequency, platform, and focus varied across partner groups in service of maximizing each partner’s expertise.
  The research team played a key role in coordinating the who, when, how, and why of co-creation activities and maintaining feedback loops.
  Clearly defining the roles of each partner type role supported process efficiency and clarity.

## Discussion

This work reports our iterative and innovative community-engaged approach to co-creating mHealth preventive care messages with and for Spanish-speaking, Arabic-speaking, and Vietnamese-speaking communities and our evaluation of engagement in the co-creation process. This is a use case of mHealth co-creation to illustrate the key ingredients of co-creation, including how our cycles of engagement and reflection informed our message design and strengthened our partnerships. We co-created culturally and linguistically appropriate messages (an average of 87 across CABs) and a delivery cadence and mode for these messages that will be embedded within our multicomponent health program. Global assessment of engagement with the co-creation process reported by our co-creation partners was high and indicated that most co-creation partners perceived the process as a collaboration or partnership, which are considered deeper forms of engagement [16].

Our study illustrates the key ingredients of co-creation that we recommend considering for community-centered design and implementation of public health programs. While it is not always feasible to include a co-creation approach from the outset of a project, we assert that the steps we endeavored may be applicable across a project lifespan (e.g., from initial design of a health program to development of the program’s evaluation). From our co-creation process, we identified several core components that expand the growing literature on co-creation and may be generalizable to other public health implementation research.[5, 17, 18] First, our engagement approach included multiple partner types and engaged them at multiple timepoints, starting from project planning and iteratively across project phases. This exemplifies the co-creation process outlined in Vargas and colleagues [5] that emphasizes active collaboration with diverse partners in an iterative and value-producing pattern. Second, the Global ARC led the hiring and supervising of the Community Weavers who, in turn, identified CAB members and led CAB meetings. The Global ARC is a trusted social change organization with established connections in the community, thus facilitating the engagement of our Community Weavers and CABs, two of our co-creation partner groups. Third, in consultation with the Global ARC, the research team (Principal Investigators and core research staff) decided that their role was primarily as observers and aids to structuring co-creation, creating space for the Community Weavers and CAB members to be primary drivers in the co-creation process. Fourth, FQHC Care Coordinators were important co-creation partners because they were familiar with both the priority communities in the study, and the FQHC culture, policies, and workflows.

The core components identified in our co-creation process are aligned with considerations for co-creation shared by Middel and colleagues [18] related to collaborator selection and dynamics. Specifically, these authors assert that engaging the appropriate partners hinges on thoughtful consideration of who will be *impacted* by the outcomes and who will be *using* the outcomes. This requires reflective thinking about which collaborators possess relevant knowledge and fostering an environment to facilitate sharing their expertise. In our project, FQHC patients and community members would be most impacted by our study outcomes related to increasing preventive health care engagement, and FQHC managers, clinicians, and staff would be those using the outcomes of our study to guide decisions about preventive health care resource allocations and clinical care. These considerations led the academic team to be mindful about the potential for unequal power dynamics among collaborators, which could negatively affect the co-creation process. This attention to power dynamics in co-creation is aligned with the principle of reflexivity, which encourages a mindful awareness of a person’s position, background, worldview and/or assumptions.[19] Elevating the Global ARC as the lead community liaison to facilitate hiring and supervising the Community Weavers, who in turn led the CABs, was an intentional element of our co-creation process to mitigate potential power imbalance and strengthen our community-driven approach.

The CAB sessions were led by Community Weavers who identified members based on their affiliation with their community and the meetings were conducted in the CAB members’ preferred languages. This required dedicated resources to language interpretation and translation to ensure that the English-speaking Global ARC facilitators, researchers, Community Weavers, and CAB members could all share in discussion. We intentionally chose co-creation activities that were conducive of bi-directional learning such as showing example message content and delivery order. We used a scaffolded and iterative approach for message creation that started with eliciting broad concerns about messages, then requesting more targeted, specific feedback on message content, language accuracy, and cultural relevance.

mHealth intervention development, while increasingly popular, has rarely used co-creation or participatory-based methods, particularly for interventions designed for underserved communities.[20, 21] Yet, co-creation processes that are inherently iterative and user-centric may lead to public health interventions that are more relevant, impactful, and sustained.[22] In our related and prior work, we developed and used a pragmatic method for tracking and documenting resources for community engagement activities.[23] We found that the most substantial investment occurred during the startup period due, in part, to the increased diversity of community engagement activities required (e.g., recruiting CAB members, coordinating the first CAB meetings, technology preparation and maintenance for CAB participation). Our experience for the current study re-affirmed the upfront but mission-critical investment required to launch our concurrent co-creation groups and activities.

Finally, although communication platforms for each partner group were critical, we learned that it was not vital that all partner groups be in direct communication using the same platform. Not every partner needed to inform the same aspects of the co-creation process, nor engage with the same frequency. The research team’s main role was to coordinate the who, when, how, why, and sequence of co-creation and ensure that information from different partners was incorporated in a timely manner, information was iteratively conveyed, and feedback loops were created. This lesson amplifies the importance of role specification and clarity among partners engaged in the co-creation process.

Our findings expand upon a growing body of research emphasizing the value of co-creation in the development of mHealth interventions. Several recent studies have documented the utility of co-creation methods in shaping culturally tailored digital health tools, particularly within underserved populations.[24] However, few have integrated community and clinical voices as extensively and iteratively as our approach. For example, Noorburgen et al. (2021)[25] and Song et al. (2021)[26] highlight the challenges of balancing feasibility and user input, often focusing more on usability testing or limited co-creation sessions rather than sustained engagement across the full co-creation cycle. Unlike previous efforts that centered primarily on end-user feedback (e.g., co-creation with patients only), our model aligned with the full spectrum of co-creation as defined by Vargas et al. [5], involving continuous collaboration among community members, clinical staff, and researchers. Furthermore, our project expands on studies such as the one by Gonzalez et al. (2021),[25, 27, 28] by implementing message review loops not only for cultural and linguistic alignment but also for real-world clinical applicability – something few studies have addressed concurrently. By embedding our process in a broader implementation framework and integrating partner input from early design through iterative testing, our approach bridges critical gaps between community-driven insight, clinical feasibility, and implementation science rigor along with potential for sustainability and transferability to other mHealth initiatives within FQHC settings.

Our study has strengths and limitations. Our primary strengths relate to the depth and breadth of our approaches to co-creation including the variety of co-creation partners engaged and the variety of activities used for engagement. The primary limitation of our study is generalizability because of our focus on Arabic, Spanish, and Vietnamese-speaking communities in a defined Southern California region. While this area, and our sample, includes an important cross-section of these cultural communities, there are expected variations in health beliefs, values, and influences across community groups. A secondary limitation is that we do not yet have evaluative data to report on the outcomes of our co-created mHealth messages. While our comprehensive and rigorous process embodying the co-creation process outlined by Vargas and colleagues [5] should facilitate mHealth content that are effective in improving preventive healthcare engagement, those impact data are not yet available. A follow-up report will be disseminated to detail outcome findings based on the final two steps (“realize” and “evaluate”) of the co-creation process [5].

From our co-created mHealth work, our next steps involve integrating our messages into our multicomponent health program that will be tested in a randomized adaptive trial.[29] We will specifically collect data using ecological momentary assessment methods to assess how well the messages and their delivery work. We will continue to meet with our CABs to evaluate the utility, relevance, and need for change of our mHealth messages to match clinical recommendations, public health guidance, and cultural context. We will also collaborate with our CABs to determine the best approaches to developing a toolkit or similar product to advance the science and practice of mHealth co-creation.

## Conclusions

This work reports a use case of mHealth co-creation to demonstrate application of a structured co-creation process. We engaged six co-creation partner groups to develop and approve mHealth preventive care messages for multilingual communities. Our co-creation illuminated several core components that expand the co-creation literature, support the increased attention to meaningful partner engagement in mHealth and public health implementation projects, and provide generalizable insights for public health implementation research.

## Supplementary Information

Below is the link to the electronic supplementary material.

Supplementary Material 1 (DOCX 85.4 KB)

## Data Availability

The datasets generated and/or analyzed during the current study are available from the corresponding author upon reasonable request.

## Abbreviations

• CAB: Community Advisory Board
• FQHC: Federally Qualified Health Center
• mHealth: mobile Health
• REST: Research Engagement Survey Tool
• WEAVE: Working towards Empowered community-driven Approaches to increase Vaccination and preventive care Engagement

## Declarations

### Clinical Trial Number

NCT05841810; Registration Date: May 3, 2023.
