# Supplementary material for: Illustrating Key Components to Co-Creation Through Preventive Care mHealth Messaging with Underserved Communities and Expert Partners
Source: J Med Syst. 2025 Dec 12;49(1):180. doi: 10.1007/s10916-025-02310-z (PMC12698742; doi:10.1007/s10916-025-02310-z)
Supplement: Supplementary file 1 — Supplementary Material 1 (DOCX 85.4 KB) [file 10916_2025_2310_MOESM1_ESM.docx]

**WEAVE STUDY mHEALTH MESSAGE LIBRARY**

**ENGLISH**

**Stage 1:** **Generic messages about missing preventive behaviors delivered to all participants**

First message:

Dear [WEAVE participant]:

This message is from San Ysidro Health. Our records show that you are missing one or more services necessary for your health. To schedule an appointment, please call (619) 662-4100.

​Second message:

You are overdue for one or more services. Taking care of your health is important to reduce your risk of disease, disability, and death. Please call or text (619) 662-4100 to schedule your appointment today.

Third message:

*Message 3 would only go to those with a continued missing vaccine. If no missing vaccines, go to Message 4.*

Our records show that you are still missing a vaccination. Receiving a vaccination can protect you and your loved ones from getting sick. Call or text 619-662-4100 to schedule your appointment today.

Fourth message:

Our records show that you are still missing a preventive care service. Getting preventive care screenings can help catch problems at an early stage. Call or text 619-662-4100 to schedule your appointment.

**Stage 2: Messages selected for each participant depending on which vaccines and preventive care behaviors are missing**

**COVID-19 vaccine**

*Group 1: co-created by study staff, CABs, expert consultants, SYH care coordinators, study staff, and clinical staff*

A. If you catch COVID, it can endanger the kids and older people in your community.

B. Choosing to get the COVID vaccine can shield both you and your loved ones from falling ill.

C. When you get vaccinated, it helps lower your risk of experiencing the harmful effects of COVID.

D. Each person who gets vaccinated helps lower the chance of their loved ones getting sick.

E. Remember, COVID isn't just a cold. It can be serious. But the vaccine can help prevent the worst outcomes. Stay safe with your shot.

F. Vaccinated people are much less likely to spread COVID to family and friends. Get your shot and be a protector, not a spreader.

G. Worried about COVID variants? The vaccine helps protect against those, too. Keep up with your shots to stay as safe as possible.

H. COVID vaccines are free and easy to get. No insurance? No problem. Let's get you vaccinated and keep you moving forward.

*Group 2: AI developed messages vetted and approved by study staff, CABs, expert consultants, SYH care coordinators, study staff, and clinical staff*

A. Every vaccination brings us closer to ending this pandemic. Do your part, get vaccinated, and let's turn the corner together.

B. Worried about COVID variants? The vaccine helps protect against those, too. Keep up with your shots to stay as safe as possible.

*(Revised Arabic) The vaccine protects you from Covid variants, follow your doses. Vaccines are free without insurance. Let us help you stay safe.*

C. Vaccinated folks are much less likely to spread COVID to family and friends. Get your shot and be a protector, not a spreader.

D. Even if you're young and healthy, COVID can surprise you with serious problems. The vaccine is your best bet for staying healthy.

E. The COVID vaccine is like a shield for your body, helping you fight off the virus if you're exposed. Strengthen your shield today!

F. Remember, COVID isn't just a cold. It can be serious. But the vaccine can help prevent the worst outcomes. Stay safe with your shot.

G. Did you know? The COVID vaccine greatly reduces your risk of ending up in the hospital. Let's keep you healthy and safe.

H. Getting the COVID vaccine helps keep our schools and workplaces open. Protect your community and keep life moving!

I. Millions have safely received the COVID vaccine. Join them, and let's get back to the things we love, safely and sooner.

**Flu vaccine**

*Group 1: co-created by study staff, CABs, expert consultants, SYH care coordinators, study staff, and clinical staff*

A. By getting the flu shot, you can reduce your chances of having to visit the doctor by 40-60%.

B. Anyone, even if they're healthy, can face serious dangers like pneumonia from the flu.

C. When you get the flu shot, you're not just safeguarding yourself but also your family.

D. Skipping the flu shot can expose vulnerable people to serious risks during flu season.

E. Flu shots are your best defense against missed work and school days due to flu illnesses. Ensure your productivity by getting your flu shot now.

F. Flu shots save lives. Studies show that flu shots significantly reduce the risk of flu-related death in children and adults.

G. Feeling healthy? You still need the flu shot. Healthy individuals can spread the flu virus without showing symptoms, risking others' health. Be a community hero.

H. Each year, the flu shot prevents millions of illnesses and flu-related doctor's visits. Join those numbers and get your flu shot today. Your health matters.

*Group 2: AI developed messages vetted and approved by study staff, CABs, expert consultants, SYH care coordinators, study staff, and clinical staff*

A. Protect your health this flu season! The flu vaccine reduces the risk of flu-associated hospitalizations among adults by about 40%. Get vaccinated today!

B. Children and the elderly are especially vulnerable to the flu. Vaccinate yourself to keep your loved ones safe. It's a simple step with a big impact.

C. Flu shots save lives. Studies show that flu vaccination significantly reduces the risk of flu-related death in children and adults. Make the responsible choice!

D. Concerned about the flu shot's effectiveness? While it varies, vaccination is still the most effective way to prevent flu and its complication. Don't wait, vaccinate!

E. Remember, the flu vaccine can't give you the flu. It's a safe way to protect yourself and your community from a serious health threat. Join the fight against flu.

F. Flu season hits hard but getting your flu vaccine is easy and often free or low cost with insurance. Check with us on how to get vaccinated without hassle.

G. Feeling healthy? You still need the flu shot. Healthy individuals can spread the flu virus without showing symptoms, risking others' health. Be a community hero.

H. Each year, the flu vaccine prevents millions of illnesses and flu-related doctor's visits. Join those numbers and get your flu shot today. Your health matters.

I. Flu vaccination is your best defense against missed work and school days due to flu illnesses. Ensure your productivity by getting your flu shot now.

J. Did you know? Getting the flu shot annually is the best way to protect against the flu viruses circulating each year. Stay up to date with your flu vaccination.

**Breast Cancer screening**

*Group 1: co-created by study staff, CABs, expert consultants, SYH care coordinators, study staff, and clinical staff*

A. Mammograms are quick, just about 20 minutes, and they can help ensure you have more quality time with your loved ones.

B. Getting regular mammograms can reduce your chances of losing your life to breast cancer.

C. Early detection through a mammogram could be a lifesaver if breast cancer is found.

D. Mammograms are the top choice for spotting breast cancer early, making treatment much simpler.

E. Did you know? Women who get regular mammograms are less likely to need aggressive treatments like surgery or chemotherapy. Keep it simple, get screened.

F. Think you're too busy for a mammogram? It takes less time than your coffee break and could give you more birthdays to celebrate.

G. Your family needs you. A quick mammogram can help make sure you're there for them, healthy and strong. Book your screening today.

H. Every woman's risk of breast cancer is different. But every woman deserves the best chance at beating it. Mammograms are that chance.

*Group 2: AI developed messages vetted and approved by study staff, CABs, expert consultants, SYH care coordinators, study staff, and clinical staff*

A. Don't wait for symptoms to appear. Mammograms can spot breast cancer early, when it's most treatable. Schedule your appointment today.

B. Breast cancer screening is all about protection. A 20-minute mammogram now could mean a world of difference later. Let's get you screened.

C. Love your life? Get a mammogram. It's a small step for a big cause: your health. Early detection means easier treatment.

D. Did you know? Women who get regular mammograms are less likely to need aggressive treatments like surgery or chemotherapy. Keep it simple, get screened.

E. Think you're too busy for a mammogram? It takes less time than your coffee break and could give you more birthdays to celebrate

F. Breast cancer doesn't always come with warning signs. That's why mammograms are crucial. They see what we can't, catching cancer early.

G. Your family needs you. A quick mammogram can help make sure you're there for them, healthy and strong. Book your screening today.

H. Mammograms save lives by finding breast cancer early when it's most treatable. Join the millions of women who get screened every year.

I. Every woman's risk of breast cancer is different. But every woman deserves the best chance at beating it. Mammograms are that chance.

J. Peace of mind is priceless. Mammograms provide that by ensuring you're taking proactive steps against breast cancer. Schedule yours.

**Colorectal cancer screening**

*Group 1: co-created by study staff, CABs, expert consultants, SYH care coordinators, study staff, and clinical staff*

A. There are different safe ways to get screened for colorectal cancer, including tests you can do at home.

B. Colorectal cancer is the third most common cancer killer, especially as you get older.

C. Screening tests for colorectal cancer are crucial because they can catch it early, when treatment works best.

D. Colorectal cancer doesn't always show signs, which is why screening is vital to catch it before it becomes a problem.

E. Prevent the preventable. Colorectal cancer can often be prevented through screening by finding and removing cells before they turn into cancer.

F. Over 45? It's time to get screened for colorectal cancer. Age increases risk, and screening is the best way to protect yourself.

G. Putting off colorectal cancer screening? Most people who get screened regret only one thing: not doing it sooner. Take control of your health.

H. Screening saves lives. People who are regularly screened for colorectal cancer have a 90% survival rate when cancer is found early.

*Group 2: AI developed messages vetted and approved by study staff, CABs, expert consultants, SYH care coordinators, study staff, and clinical staff*

A. Colorectal cancer screening could save your life. It's simple and there are several options, including easy tests you can do at home. Don't wait, get screened!

B. Did you know? Screening can find colorectal cancer early when it's most treatable. Early detection makes all the difference. Schedule your screening today.

C. You might feel fine and still have colorectal cancer. Screening helps find it early, even without symptoms. It's a step you can take for peace of mind.

D. Prevent the preventable. Colorectal cancer can often be prevented through screening by finding and removing polyps before they turn into cancer.

E. Over 45? It's time to get screened for colorectal cancer. Age increases risk, and screening is the best way to protect yourself.

F. Putting off colorectal cancer screening? Most people who get screened regret only one thing: not doing it sooner. Take control of your health.

G. Colorectal cancer screening is more than a test—it's a way to take charge of your health. Many insurance plans cover it, making now the perfect time.

H. Worried about discomfort? Modern screening options are more comfortable than ever. Some can be done entirely at home, at your convenience.

I. Screening saves lives. People who are regularly screened for colorectal cancer have a 90% survival rate when cancer is found early.

J. Join the millions who've taken steps to prevent colorectal cancer. Screening is one of the most powerful tools we have. Let's use it and stay healthy together.

**Cervical cancer screening**

*Group 1: co-created by study staff, CABs, expert consultants, SYH care coordinators, study staff, and clinical staff*

A. The Pap test helps find any “precancer” cells, so it can catch cervical cancer before it starts.

B. By making a Pap test part of your healthcare routine, you're taking proactive steps to safeguard against cervical cancer.

C. Access to Pap tests plays a vital role in reducing cervical cancer fatalities.

D. Since cervical cancer often shows no signs in its initial phases, screening is crucial for catching it early when treatment is most effective.

E. Early detection is key to beating cervical cancer. Regular Pap tests can catch it before it starts. Take charge of your health with a quick check-up.

F. Did you know? Cervical cancer deaths have significantly decreased thanks to Pap tests. Be part of this success story. Get screened.

G. Protect your future with a Pap test. It's one of the most effective ways to prevent cervical cancer. Empower yourself with regular screenings.

H. Be a health hero. Regular Pap tests can catch cervical cancer early, making treatment easier and more effective. Book your screening appointment now.

*Group 2: AI developed messages vetted and approved by study staff, CABs, expert consultants, SYH care coordinators, study staff, and clinical staff*

A. A simple Pap test could save your life by detecting cervical cancer early. Don't wait for symptoms to appear. Schedule your screening today.

B. Early detection is key to beating cervical cancer. Regular Pap tests can catch it before it starts. Take charge of your health with a quick check-up.

C. Pap tests are quick, easy, and could be lifesavers. Join the millions of women who get screened regularly and protect yourself against cervical cancer.

D. Cervical cancer doesn't have to be a threat. Regular Pap tests can spot problems early when they're most treatable. Make screening a part of your health routine.

E. Did you know? Cervical cancer fatalities have significantly decreased thanks to Pap tests. Be part of this success story. Get screened.

F. No symptoms? You still need a Pap test. Cervical cancer can be silent but deadly. Early screening is your best defense.

G. Taking just a few minutes for a Pap test every few years can make a lifetime of difference. Prioritize your health. Schedule your test.

H. A Pap test does more than screen for cancer; it offers peace of mind. Knowing you're taking proactive steps for your health is invaluable.

I. Protect your future with a Pap test. It's one of the most effective ways to prevent cervical cancer. Empower yourself with regular screenings.

J. Be a health hero. Regular Pap tests can catch cervical cancer early, making treatment easier and more effective. Book your screening appointment now.

**SPANISH**

**Stage 1:** **Generic messages about missing preventive behaviors delivered to all participants**

Primer mensaje:

Estimado [weave_participant_arm_1][name],

Este mensaje es de San Ysidro Health. Nuestros registros muestran que le faltan uno o más servicios de salud necesarios. Para programar una cita, por favor llame al (619) 662-4100

Second message:
Usted esta demorado para uno o más servicios. Un adecuado cuidado de la salud es importante para reducir el riesgo de enfermedades, discapacidad y/o muerte. Favor de llamar al (619) 662-4100 para agendar su cita hoy.

Third message:
Nuestra base de datos muestra que a usted todavía le falta una vacuna. Al recibir una vacuna, usted se protege a usted y a su familia. Favor de llamar por teléfono al 619-662-4100 para agendar su cita hoy.

Fourth message:

Nuestra base de datos muestra que a usted todavía le falta cuidado preventivo. Recibiendo cuidado preventivo de exámenes médicos puede ayudar captar problemas en una etapa temprana. Favor de llamar por teléfono al 619-662-4100 para agendar su cita hoy.

**Stage 2: Messages selected for each participant depending on which vaccines and preventive care behaviors are missing**

**COVID-19 vaccine**

1. Si a usted le da COVID-19, puede poner en peligro a los niños y a personas mayores en su comunidad. (A1)
2. La vacuna contra el COVID es como un escudo para tu cuerpo, que te ayuda a combatir el virus si estás expuesto. ¡Fortalece tu escudo hoy! (E2)
3. Cada persona que se vacuna ayuda a prevenir el riesgo de enfermedad a sus seres queridos. (D1)
4. ¿Preocupado por las variantes de COVID? La vacuna también ayuda a proteger contra ellos. Manténgase al día con sus vacunas para mantenerse lo más seguro posible. (B2/G1)
5. Cuando usted se vacuna, ayuda a disminuir el riesgo de efectos letales por COVID-19. (C1)
6. Vacunarse contra el COVID ayuda a mantener abiertas nuestras escuelas y lugares de trabajo. Proteja a su comunidad y mantenga la vida en movimiento. (H2)
7. Las vacunas COVID son gratuitas y fáciles de obtener. ¿Sin seguro? Ningún problema. Vacunémoslo y sigamos avanzando. (H1)
8. ¿Sabías que...? La vacuna contra el COVID reduce en gran medida el riesgo de terminar en el hospital. Mantengámoste sano y seguro. (G2)
9. El colocarse la vacuna contra COVID-19 puede protegerlo a usted y a sus seres queridos de enfermedad. (B1)
10. Las personas vacunadas tienen muchas menos probabilidades de transmitir COVID-19 a familiares y amigos. Reciba su vacuna y sea un protector, no un esparcidor. (F1/C2)
11. Incluso si eres joven y saludable, el COVID puede sorprenderte con problemas graves. La vacuna es su mejor opción para mantenerse saludable. (D2)
12. Recuerde, COVID-19 no solamente es un resfriado. Puede ser serio. La vacuna puede ayudar a prevenir situaciones peores. Manténgase sano con su vacuna. (E1/F2)
13. Millones de personas han recibido la vacuna contra el COVID de forma segura. Únase a ellos y volvamos a las cosas que amamos, de manera segura y más pronto. (I2)
14. Cada vacuna nos acerca más a poner fin a esta pandemia. Haz tu parte, vacúnate y demos la vuelta a la esquina juntos. (A2)

| **Targeted Messages Phase 1** | | | | | | | | | | | | | | | | | | | |
| --- | --- | --- | --- | --- | --- | --- | --- | --- | --- | --- | --- | --- | --- | --- | --- | --- | --- | --- | --- |
| COVID | Spanish^*^ | | A(1) | | D(3) | | C(5) | | H(7) | | B(9) | | | G(4) | | E(12) | | F(10) | |
| **Targeted Messages Phase 2** | | | | | | | | | | | | | | | | | | | |
| COVID | Spanish | E(2) | | B(4) | | H(6) | | G(8) | | C(10) | | D(11) | I(13) | | F(12) | | A(14) | |  |

*People thought C and D could be combined into one message

General notes:

· Spanish CAB liked question-oriented messages

· Shorter is better, not a lot of words (Spanish)

· Can CAB members receive the messages and want to know when study gets started?

**Flu vaccine**

1. Las vacunas contra la gripe salvan vidas. Los estudios muestran que las vacunas contra la gripe reducen significativamente el riesgo de muerte relacionada con la gripe en niños y adultos. (F1/C2)
2. Cada año, la vacuna contra la gripe previene millones de enfermedades y visitas al médico relacionadas con la gripe. Únase a esos números y vacúnese contra la gripe hoy. Tu salud importa. (H1/H2)
3. La temporada de gripe golpea fuerte, pero vacunarse contra la gripe es fácil y, a menudo, gratis o de bajo costo con seguro médico. Consulte con nosotros cómo vacunarse sin problemas. (F2)
4. Saltarse la vacuna contra la gripe puede exponer a las personas vulnerables a riesgos graves durante la temporada de gripe. (D1)
5. ¿Le preocupa la eficacia de la vacuna contra la gripe? Si bien varía, la vacunación sigue siendo la forma más eficaz de prevenir la influenza y sus complicaciones. ¡No esperes, vacúnate! (D2)
6. Cuando se vacuna contra la gripe, no sólo se protege a sí mismo sino también a su familia. (C1)
7. Los niños y los ancianos son especialmente vulnerables a la gripe. Vacúnese para mantener seguros a sus seres queridos. Es un paso sencillo con un gran impacto. (B2)
8. ¡Proteja su salud esta temporada de gripe! La vacuna contra la influenza reduce el riesgo de hospitalizaciones asociadas a la influenza entre los adultos en aproximadamente un 40 %. ¡Vacúnese hoy! (A1)
9. Al vacunarse contra la gripe, puede reducir sus posibilidades de tener que visitar al médico entre un 40 y un 60 %.(A2)
10. Cualquier persona, incluso si está sana, puede enfrentarse a peligros graves, como la neumonía por gripe.(B1)
11. ¿Se siente saludable? Aún necesitas la vacuna contra la gripe. Las personas sanas pueden transmitir el virus de la gripe sin mostrar síntomas, poniendo en riesgo la salud de los demás. Sea un héroe comunitario. (G1/G2)
12. Recuerde que la vacuna contra la gripe no puede contagiarle la gripe. Es una forma segura de protegerse a sí mismo y a su comunidad de una amenaza grave para la salud. Únete a la lucha contra la gripe. (E2)
13. Las vacunas contra la gripe son su mejor defensa contra los días laborales y escolares perdidos debido a enfermedades gripales. Asegure su productividad vacunándose contra la gripe ahora. (E1/I2)
14. ¿Sabías que...? Vacunarse contra la gripe anualmente es la mejor manera de protegerse contra los virus de la gripe que circulan cada año. Manténgase al día con su vacuna contra la gripe. (J2)

| **Targeted Messages Phase 1** | | | | | | | | | | | | | | | | | | |
| --- | --- | --- | --- | --- | --- | --- | --- | --- | --- | --- | --- | --- | --- | --- | --- | --- | --- | --- |
| FLU | Spanish | F(1) | | H(2) | | D(4) | | C(6) | | A(8) | | | B(10) | | G(11) | | E(13) | |
| **Targeted Messages Phase 2** | | | | | | | | | | | | | | | | | | |
| FLU | Spanish | H(2) | F(3) | | D(5) | | B(7) | | A(9) | | C(1) | E(12) | | I(13) | | G(11) | | J(14) |

**Breast Cancer screening**

1. La detección temprana a través de una mamografía salvar la vida si el cáncer de seno es detectado a tiempo. (C1)
2. ¿Sabía usted que? Las mujeres que se hacen mamografías periódicamente tienen menos probabilidades de necesitar tratamientos agresivos como cirugía o quimioterapia. Manténgalo simple, hágase la prueba. (D2/E1)
3. ¿Esta muy ocupada para realizarse una mamografía? Toma menos tiempo que preparar el café y le puede garantizar muchos cumpleaños más. (F1/E2)
4. El cáncer de mama no siempre viene acompañado de señales de advertencia. Es por eso que las mamografías son cruciales. Ellos ven lo que nosotros no podemos, detectar el cáncer a tiempo. (F2)
5. El cáncer de seno puede aparecer en cualquier parte de la mama. Al realizarse una mamografía, se puede detectar la zona y otorgar tratamiento oportuno. (H1/I2)
6. Su familia la necesita, agende una mamografía hoy y regale más momentos de calidad. (G1/G2)
7. No esperes a que aparezcan los síntomas. Las mamografías pueden detectar el cáncer de mama en sus etapas iniciales, cuando es más tratable. Programe su cita hoy mismo. (A2)
8. Las mamografías son rápidas, solo duran unos 20 minutos, y pueden ayudar a garantizar que usted pase más tiempo de calidad con sus seres queridos. (A1)
9. La tranquilidad no tiene precio. Las mamografías proporcionan eso al garantizar que estés tomando medidas proactivas contra el cáncer de mama. Programe el suyo. (J2)
10. Las mamografías son la mejor opción para detectar el cáncer de mama en sus etapas iniciales, lo que simplifica mucho el tratamiento. (D1)
11. Las pruebas de detección del cáncer de mama tienen que ver con la protección. Una mamografía de 20 minutos ahora podría significar una gran diferencia más adelante. Vamos a hacerte una prueba. (B2)
12. Al realizarse exámenes regulares de mamografías, puede reducir sus probabilidades de perder su vida contra el cáncer de seno. (B1)
13. ¿Amas tu vida? Hazte una mamografía. Es un pequeño paso para una gran causa: tu salud. La detección temprana significa un tratamiento más fácil. (C2)
14. Las mamografías salvan vidas al detectar el cáncer de mama temprano, cuando es más tratable. Únase a los millones de mujeres que se someten a pruebas de detección cada año. (H2)

| **Targeted Messages Phase 1** | | | | | | | | | | | | | | | | | | | |
| --- | --- | --- | --- | --- | --- | --- | --- | --- | --- | --- | --- | --- | --- | --- | --- | --- | --- | --- | --- |
| Mammogram | Spanish | | C(1) | | F(3) | | E(2) | | G(6) | | H(5) | | | A(8) | | D(10) | | B(12) | |
| **Targeted Messages Phase 2** | | | | | | | | | | | | | | | | | | | |
| Mammogram | Spanish | D(2) | | F(4) | | I(5) | | A(7) | | E(3) | | G(6) | J(9) | | B(11) | | C(13) | | H(14) |

**Colorectal cancer screening**

1. ¿Mayor de 45? Es tiempo para revisarse contra el cáncer colorrectal. La edad aumenta el riesgo, y revisándose con su Doctor es la manera mejor para protegerse. (F1/E2)
2. La detección del cáncer colorrectal es más que una prueba: es una forma de hacerse cargo de su salud. Muchos planes de seguro lo cubren. (G2)
3. El cáncer colorrectal no siempre aparece con síntomas, es por ello que es vital hacerse pruebas rutinarias. (D1)
4. Las pruebas de detección del cáncer colorrectal podrían salvarle la vida. Es simple y hay varias opciones, incluidas pruebas fáciles que puedes hacer en casa. ¡No esperes, hazte una prueba. (A2)
5. Las pruebas para revisar el cáncer colorrectal son cruciales porque pueden detectarlo temprano cuando el tratamiento funciona mejor. (C1)
6. Es posible que se sienta bien y aún tenga cáncer colorrectal. Las pruebas de detección ayudan a detectarla a tiempo, incluso sin síntomas. Es un paso que puedes dar para estar tranquilo. (C2)
7. Revisarse salva vidas. Personas que regularmente se revisan para el cáncer colorrectal tienen un 90% de sobrevivir cuando el cáncer se encuentra temprano. (H1/I2)
8. Hay diferentes maneras seguras de hacer la prueba para el cáncer colorrectal, incluyendo pruebas que puede hacer en casa. (A1)
9. Prevenir lo prevenible. El cáncer colorrectal a menudo se puede prevenir mediante exámenes de detección mediante la detección y extirpación de pólipos antes de que se conviertan en cáncer. (D2)
10. El cáncer colorrectal es el tercer cáncer más común de muertes relacionadas por cáncer, especialmente si es usted mayor de edad. (B1)
11. ¿Sabías que...? Las pruebas de detección pueden detectar el cáncer colorrectal en una etapa temprana, cuando es más tratable. La detección temprana marca la diferencia. Programe su proyección hoy mismo. (B2)
12. Prevenir lo prevenible. El cáncer colorrectal a menudo se puede prevenir mediante pruebas de detección, al encontrar y eliminar células antes de que se conviertan en cáncer. (E1)
13. ¿Te preocupa la incomodidad? Las opciones de cribado modernas son más cómodas que nunca. Algunos se pueden hacer completamente en casa, a su conveniencia. (H2)
14. ¿Posponer el estudio de cáncer colorrectal? Muchas de las personas que son detectas con cáncer colorrectal se arrepienten de una cosa: No hacerlo antes. Tome control de su salud. (G1/F2)
15. Únase a los millones de personas que han tomado medidas para prevenir el cáncer colorrectal. El cribado es una de las herramientas más poderosas que tenemos. Tomemos uso de el y mantengámonos sanos juntos. (J2)

| **Targeted Messages Phase 1** | | | | | | | | | | | | | | | | | | | |
| --- | --- | --- | --- | --- | --- | --- | --- | --- | --- | --- | --- | --- | --- | --- | --- | --- | --- | --- | --- |
| Colorectal | Spanish | | F(1) | | D(3) | | C(5) | | H(7) | | A(8) | | | B(10) | | E(12) | | G(14) | |
| **Targeted Messages Phase 2** | | | | | | | | | | | | | | | | | | | |
| Colorectal | Spanish | G(2) | | A(4) | | C(6) | | E(1) | | D(9) | | B(11) | H(13) | | F(14) | | I(7) | | J(15) |

**Cervical cancer screening**

1. ¿Sabías que...? Las muertes por cáncer de cuello uterino han disminuido significativamente gracias a las pruebas de Papanicolaou. Sé parte de esta historia de éxito. Hágase una prueba de detección. (F1/E2)
2. Proteja su futuro con una prueba de Papanicolaou. Es una de las formas más efectivas de prevenir el cáncer de cuello uterino. Empodérate con exámenes regulares. (G1/I2)
3. El cáncer cervicouterino muchas veces se presenta asintomático. Una prueba, podría salvar su vida. (D1)
4. La detección temprana, es elemental contra el cáncer cervicouterino. Una prueba de Papanicolaou puede ser la diferencia. Agende la suya hoy mismo. (E1/B2)
5. Las pruebas de Papanicolaou son rápidas, fáciles y pueden salvarle la vida. Únase a los millones de mujeres que se someten a pruebas de detección con regularidad y protéjase contra el cáncer de cuello uterino. (C2)
6. Una prueba de Papanicolaou hace más que detectar el cáncer; Ofrece tranquilidad. Saber que está tomando medidas proactivas para su salud es invaluable. (H2)
7. El acceso a pruebas de Papanicolau permite reducir de manera considerable los casos de cáncer cervicouterino. (C1)
8. ¿No hay síntomas? Todavía necesita una prueba de Papanicolaou. El cáncer de cuello uterino puede ser silencioso pero mortal. La detección temprana es su mejor defensa. (F2)
9. Las pruebas de Papanicolaou ayudan a detectar células precancerosas. Al detectarlas, se puede evitar que estas se conviertan en cáncer cervicouterino. (A1)
10. Tomarse solo unos minutos para una prueba de Papanicolaou cada pocos años puede marcar la diferencia de por vida. Prioriza tu salud. Programe su prueba. (G2)
11. Al realizarse una prueba de Papanicolaou, usted esta tomando un gran paso contra el cáncer cervicouterino. (B1)
12. Sea un héroe de salud. Las pruebas de Papanicolaou pueden detectar el cáncer cervicouterino temprano, haciendo el tratamiento fácil y más efectivo. Agende su cita hoy. (H1/J2)
13. El cáncer de cuello uterino no tiene por qué ser una amenaza. Las pruebas de Papanicolaou regulares pueden detectar problemas temprano, cuando son más tratables. Haga que las pruebas de detección formen parte de su rutina de salud. (D2)
14. Una simple prueba de Papanicolaou podría salvarle la vida al detectar el cáncer de cuello uterino a tiempo. No esperes a que aparezcan los síntomas. Programe su proyección hoy mismo. (A2)

| **Targeted Messages Phase 1** | | | | | | | | | | | | | | | | | | | |
| --- | --- | --- | --- | --- | --- | --- | --- | --- | --- | --- | --- | --- | --- | --- | --- | --- | --- | --- | --- |
| PAP | Spanish | | F(1) | | D(3) | | E(4) | | G(1) | | C(7) | | | A(9) | | B(11) | | H(12) | |
| **Targeted Messages Phase 2** | | | | | | | | | | | | | | | | | | | |
| PAP | Spanish | I(2) | | B(4) | | C(5) | | H(6) | | F(8) | | G(10) | E(1) | | D(13) | | J(12) | | A(14) |

**VIETNAMESE**

**Stage 1:** **Generic messages about missing preventive behaviors delivered to all participants**

1) Tin nhắn đầu tiên:

Kính thưa [weave_participant_arm_1][name],

Tin nhắn này là từ San Ysidro Health. Hồ sơ của chúng tôi cho thấy rằng bạn đang thiếu một hoặc nhiều dịch vụ y tế cần thiết. Để đặt lịch hẹn, vui lòng gọi (619) 662-4100.

2) Tin nhắn thứ hai:

Bạn đã quá hạn cho một hoặc nhiều dịch vụ. Chăm sóc sức khỏe của bạn là yếu tố quan trọng để giảm nguy cơ mắc bệnh, khuyết tật và tử vong. Vui lòng gọi hoặc nhắn tin (619) 662-4100 để đặt lịch hẹn ngay hôm nay.

3) Tin nhắn thứ ba:

*Thông điệp 3 sẽ chỉ dành cho những người vẫn tiếp tục chưa lấy hẹn tiêm vắc xin. Nếu không bị thiếu vắc xin, chuyển tới Tin nhắn 4.*

Hồ sơ của chúng tôi cho thấy bạn vẫn chưa được tiêm chủng. Tiêm vắc-xin có thể bảo vệ bạn và những người thân yêu của bạn khỏi bị bệnh. Gọi hoặc nhắn tin tới số 619-662-4100 để đặt lịch hẹn ngay hôm nay.

4) Tin nhắn thứ tư:

Hồ sơ của chúng tôi cho thấy rằng bạn vẫn đang thiếu dịch vụ chăm sóc phòng ngừa. Việc sàng lọc chăm sóc phòng ngừa có thể giúp phát hiện các vấn đề ở giai đoạn đầu. Gọi hoặc nhắn tin tới số 619-662-4100 để đặt lịch hẹn.

**Stage 2: Messages selected for each participant depending on which vaccines and preventive care behaviors are missing**

**COVID-19 vaccine**/ **Vắc-xin phòng ngừa covid-19**

1. Vắc xin ngừa COVID được cung cấp miễn phí và dễ dàng nhận được. Không có bảo hiểm? Không có vấn đề gì. Hãy tiêm phòng cho bạn và giúp bạn tiến về phía trước. (H1)
2. Ngay cả khi bạn còn trẻ và khỏe mạnh, COVID có thể khiến bạn bất ngờ với những vấn đề nghiêm trọng. Vắc-xin là lựa chọn tốt nhất để bạn luôn khỏe mạnh. (D2)
3. Hãy nhớ rằng, COVID không chỉ là cảm lạnh. Nó có thể nghiêm trọng. Nhưng vắc-xin có thể giúp ngăn ngừa những kết quả tồi tệ nhất. Giữ an toàn cho bạn bằng mũi tiêm chủng. (E1/F2)
4. Nếu bạn mắc phải COVID, nó có thể gây nguy hiểm cho trẻ em và người lớn tuổi trong cộng đồng của bạn. (A1)
5. Mỗi lần tiêm chủng đều đưa chúng ta đến gần hơn với việc chấm dứt đại dịch này. Hãy làm phần việc của mình, tiêm phòng và cùng nhau vượt qua khó khăn. (A2)
6. Việc chọn tiêm vắc xin COVID có thể bảo vệ bạn và những người thân yêu của bạn khỏi bị bệnh. (B1)
7. Lo lắng về các biến thể COVID? Vắc-xin cũng giúp bảo vệ chống lại những biến chủng đó. Tiêm đủ các mũi tiêm cho bạn để giữ an toàn nhất có thể. (G1/B2)
8. Khi bạn tiêm vắc-xin, nó sẽ giúp bạn giảm nguy cơ đối đầu với những tác hại của COVID. (C1)
9. Những người được tiêm chủng ít có khả năng lây lan COVID cho gia đình và bạn bè hơn nhiều. Hãy tiêm chủng và trở thành người bảo vệ cộng đồng chứ không phải người truyền bệnh. (F1/C2)
10. Mỗi người được tiêm chủng sẽ giúp giảm nguy cơ người thân của họ mắc bệnh. (D1)
11. Vắc xin ngừa COVID giống như một tấm lá chắn cho cơ thể bạn, giúp bạn chống lại vi rút nếu bị phơi nhiễm. Hãy tăng cường lá chắn của bạn ngay hôm nay! (E2)
12. Bạn có biết không? Vắc xin COVID làm giảm đáng kể nguy cơ bạn phải nhập viện. Hãy giữ cho bạn khỏe mạnh và an toàn. (G2)
13. Việc tiêm vắc xin COVID giúp trường học và nơi làm việc của chúng ta tiếp tục mở cửa. Bảo vệ cộng đồng của bạn và giữ cho cuộc sống luôn vận động. (H2)
14. Hàng triệu người đã được tiêm vắc xin ngừa Covid một cách an toàn. Hãy tham gia cùng họ và quay lại với những điều chúng ta yêu thích một cách an toàn và sớm hơn. (I2)

| **Targeted Messages Phase 1** | | | | | | | | | | | | | | | | | | | |  |
| --- | --- | --- | --- | --- | --- | --- | --- | --- | --- | --- | --- | --- | --- | --- | --- | --- | --- | --- | --- | --- |
| COVID | Vietnamese | | H(1) | | E(3) | | A(4) | | B(6) | | C(8) | | | D(10) | | F(9) | | G(7) | |  |
| **Targeted Messages Phase 2** | | | | | | | | | | | | | | | | | | | | |
| COVID | Vietnamese^2^ | D(2) | | F(3) | | A(5) | | B(7) | | C(9) | | E(11) | G(12) | | H(13) | | I(14) | |  | |

2- The first two messages are enough (D and F). The rest are superfluous - people won’t read a lot of text messages

**Flu vaccine/** **Vắc-xin cúm**

1. Bạn đang cảm thấy rấkhỏe mạnh? Bạn vẫn cần tiêm phòng cúm. Những người khỏe mạnh có thể lây lan vi-rút cúm mà không biểu hiện triệu chứng, gây nguy hiểm cho sức khỏe của người khác. Hãy là một anh hùng cộng đồng. (G1/G2)
2. Hãy nhớ rằng vắc xin cúm không thể khiến bạn bị cúm. Đó là một cách an toàn để bảo vệ bản thân và cộng đồng của bạn khỏi mối đe dọa sức khỏe nghiêm trọng. Tham gia cuộc chiến chống lại bệnh cúm. (E2)
3. Bằng cách tiêm phòng cúm, bạn có thể giảm 40-60% khả năng phải đến gặp bác sĩ. (A1)
4. Tiêm phòng cúm cứu mạng sống. Các nghiên cứu cho thấy tiêm phòng cúm làm giảm đáng kể nguy cơ tử vong do cúm ở trẻ em và người lớn. (F1/C2)
5. Bất cứ ai, ngay cả khi khỏe mạnh, cũng có thể phải đối mặt với những mối nguy hiểm nghiêm trọng như viêm phổi do cúm. (B1)
6. Hãy bảo vệ sức khỏe của bạn trong mùa cúm này! Vắc-xin cúm làm giảm khoảng 40% nguy cơ nhập viện do cúm ở người lớn. Hãy tiêm phòng ngay hôm nay! (A2)
7. Khi tiêm phòng cúm, bạn không chỉ bảo vệ bản thân mà còn cho gia đình mình. (C1)
8. Trẻ em và người già đặc biệt dễ bị cúm. Hãy chủng ngừa cho chính mình để giữ an toàn cho những người thân yêu của bạn. Đó là một bước đơn giản nhưng có tác động lớn. (B2)
9. Bỏ qua việc tiêm phòng cúm có thể khiến những người dễ bị tổn thương gặp rủi ro nghiêm trọng trong mùa cúm. (D1)
10. Lo ngại về hiệu quả của việc tiêm phòng cúm? Mặc dù hiệu quả của nó có nhiều khác biệt nhưng tiêm chủng vẫn là cách hiệu quả nhất để ngăn ngừa bệnh cúm và các biến chứng của bệnh. Đừng chờ đợi, hãy tiêm phòng! (D2)
11. Tiêm phòng cúm là biện pháp phòng ngừa tốt nhất để bạn không phải nghỉ làm và nghỉ học do bệnh cúm. Đảm bảo năng suất của bạn bằng cách tiêm phòng cúm ngay bây giờ. (E1/I2)
12. Mùa cúm đang đến gần nhưng việc tiêm vắc-xin cúm rất dễ dàng và thường miễn phí hoặc chi phí thấp nếu có bảo hiểm. Hãy cùng chúng tôi tìm hiểu cách tiêm vắc-xin mà không gặp rắc rối. (F2)
13. Mỗi năm, mũi tiêm phòng cúm ngăn ngừa hàng triệu bệnh tật và việc phải đi khám bác sĩ liên quan đến cúm. Hãy tăng những con số đó lên và tiêm phòng cúm ngày hôm nay. Sức khỏe của bạn rất quan trọng. (H1/H2)
14. Bạn có biết không? Tiêm phòng cúm hàng năm là cách tốt nhất để bảo vệ chống lại vi-rút cúm lưu hành hàng năm. Luôn cập nhật về việc tiêm phòng cúm của bạn. (J2)

| **Targeted Messages Phase 1** | | | | | | | | | | | | | | | | | | | |  |
| --- | --- | --- | --- | --- | --- | --- | --- | --- | --- | --- | --- | --- | --- | --- | --- | --- | --- | --- | --- | --- |
| Flu | Vietnamese | | G(1) | | A(3) | | B(5) | | C(7) | | D(9) | | | E(11) | | F(4) | | H(13) | |  |
| **Targeted Messages Phase 2** | | | | | | | | | | | | | | | | | | | | |
| Flu | Vietnamese | E(2) | | C(4) | | A(6) | | B(8) | | D(10) | | F(12) | G(1) | | H(13) | | I(11) | | J(14) | |

**Breast Cancer screening/ Sàng lọc ung thư vú**

1. Chụp quang tuyến vú là lựa chọn hàng đầu để phát hiện sớm ung thư vú, giúp việc điều trị trở nên đơn giản hơn nhiều. (D1)
2. Ung thư vú không phải lúc nào cũng có dấu hiệu cảnh báo. Đó là lý do tại sao chụp quang tuyến vú là rất quan trọng. Chụp quang tuyến vú giúp nhìn thấy những gì chúng ta không thể, phát hiện sớm bệnh ung thư. (F2)
3. Chụp X-quang tuyến vú diễn ra nhanh chóng, chỉ khoảng 20 phút và chúng có thể giúp đảm bảo bạn có nhiều thời gian chất lượng hơn với những người thân yêu của mình. (A1)
4. Đừng chờ đợi các triệu chứng xuất hiện. Chụp quang tuyến vú có thể phát hiện sớm ung thư vú, khi bệnh có khả năng điều trị tốt nhất. Hãy đặt lịch hẹn ngày hôm nay. (A2)
5. Chụp quang tuyến vú thường xuyên có thể làm giảm nguy cơ tử vong vì ung thư vú. (B1)
6. Sàng lọc ung thư vú là để bảo vệ. Chụp quang tuyến vú trong 20 phút bây giờ có thể có nghĩa là một thế giới khác biệt sau này. Hãy để bạn được sàng lọc. (B2)
7. Phát hiện sớm thông qua chụp quang tuyến vú có thể là cứu cánh nếu phát hiện ung thư vú. (C1)
8. Bạn có yêu cuộc sống của bạn? Hãy chụp quang tuyến vú. Đó là một bước nhỏ cho một mục đích lớn: sức khỏe của bạn. Phát hiện sớm giúp điều trị dễ dàng hơn. (C2)
9. Bạn có biết không? Những phụ nữ chụp quang tuyến vú thường xuyên ít có khả năng cần các phương pháp điều trị tích cực như phẫu thuật hoặc hóa trị. Hãy làm cho mọi việc đơn giản hơn bằng cách chọn làm sàng lọc sớm. (E1/D2)
10. Nghĩ rằng bạn quá bận rộn để chụp quang tuyến vú? Việc này tốn ít thời gian hơn thời gian nghỉ uống cà phê và có thể mang lại cho bạn nhiều sinh nhật hơn để ăn mừng. (F1/E2)
11. Gia đình bạn cần bạn. Chụp quang tuyến vú nhanh có thể giúp đảm bảo rằng bạn luôn ở bên họ, khỏe mạnh và mạnh mẽ. Hãy đặt hẹn để làm chụp quang ngay hôm nay. (G1/G2)
12. Chụp quang tuyến vú có thể cứu mạng sống bằng cách phát hiện sớm bệnh ung thư vú khi bệnh có thể điều trị được tốt nhất. Hãy tham gia cùng hàng triệu phụ nữ được sàng lọc hàng năm. (H2)
13. Nguy cơ mắc bệnh ung thư vú của mỗi phụ nữ là khác nhau. Nhưng mọi phụ nữ đều xứng đáng có cơ hội tốt nhất để đánh bại nó. Chụp quang tuyến vú là cơ hội đó. (H1/I2)
14. Sự yên tâm là vô giá. Chụp quang tuyến vú mang lại điều đó bằng cách đảm bảo bạn thực hiện các bước chủ động chống lại bệnh ung thư vú. Hãy lên lịch cho bạn. (J2)

| **Targeted Messages Phase 1** | | | | | | | | | | | | | | | | | |  |
| --- | --- | --- | --- | --- | --- | --- | --- | --- | --- | --- | --- | --- | --- | --- | --- | --- | --- | --- |
| Mammogram | Vietnamese^1^ | | D(1) + A(3) | | | B(5) | | C(7) | | E(9) | | F(10) | | G(11) | | H(13) | |  |
| **Targeted Messages Phase 2** | | | | | | | | | | | | | | | | | | |
| Mammogram | Vietnamese | F(2) | | A(4) | B(6) | | C(8) | | D(9) | | E(10) | G(11) | H(12) | | I(13) | | J(14) | |

1 People thought that D and A should be combined into one message

**Colorectal cancer screening/ Sàng lọc đại trực tràng**

1. Trên 45 tuổi? Đã đến lúc phải tầm soát ung thư đại trực tràng. Tuổi tác làm tăng nguy cơ và sàng lọc là cách tốt nhất để bảo vệ bạn. (F1/E2)
2. Tầm soát ung thư đại trực tràng không chỉ là một xét nghiệm - đó là một cách để kiểm soát sức khỏe của bạn. Nhiều chương trình bảo hiểm chi trả cho việc này. (G2)
3. Sàng lọc cứu sống bạn. Những người thường xuyên được tầm soát ung thư đại trực tràng có tỷ lệ sống sót là 90% khi ung thư được phát hiện sớm. (H1/I2)
4. Sàng lọc ung thư đại trực tràng có thể cứu sống bạn. Việc này rất đơn giản và có một số lựa chọn, bao gồm cả những cách kiểm tra dễ dàng mà bạn có thể thực hiện tại nhà. Đừng chờ đợi, hãy sàng lọc! (A2)
5. Hãy phòng ngừa những gì có thể phòng ngừa được. Ung thư đại trực tràng thường có thể được ngăn ngừa thông qua sàng lọc bằng cách tìm và loại bỏ các tế bào trước khi chúng chuyển thành ung thư. (E1)
6. Trì hoãn sàng lọc ung thư đại trực tràng? Hầu hết những người được sàng lọc đều hối tiếc một điều: không làm điều đó sớm hơn. Hãy kiểm soát sức khỏe của bạn. (G1/F2)
7. Có nhiều cách an toàn khác nhau để sàng lọc ung thư đại trực tràng, bao gồm các xét nghiệm bạn có thể thực hiện tại nhà. (A1)
8. Bạn có biết không? Sàng lọc có thể phát hiện sớm ung thư đại trực tràng khi bệnh có khả năng điều trị tốt nhất. Phát hiện sớm sẽ tạo nên sự khác biệt. Hãy lên lịch để thực hiện sàng lọc sớm ngày hôm nay. (B2)
9. Ung thư đại trực tràng là nguyên nhân gây ung thư phổ biến thứ ba, đặc biệt là khi bạn có tuổi. (B1)
10. Bạn có thể cảm thấy ổn nhưng vẫn có thể bị ung thư đại trực tràng. Sàng lọc giúp phát hiện sớm, thậm chí không có triệu chứng. Đó là một bước bạn có thể thực hiện để yên tâm. (C2)
11. Các xét nghiệm sàng lọc ung thư đại trực tràng rất quan trọng vì chúng có thể phát hiện sớm bệnh khi phương pháp điều trị có hiệu quả tốt nhất. (C1)
12. Phòng ngừa điều có thể phòng ngừa được. Ung thư đại trực tràng thường có thể được ngăn ngừa thông qua sàng lọc bằng cách tìm và loại bỏ polyp trước khi chúng chuyển thành ung thư. (D2)
13. Ung thư đại trực tràng không phải lúc nào cũng có dấu hiệu, đó là lý do tại sao việc sàng lọc là rất quan trọng để phát hiện bệnh trước khi nó trở thành vấn đề. (D1)
14. Lo lắng về sự khó chịu? Các lựa chọn sàng lọc hiện đại thoải mái hơn bao giờ hết. Một số có thể được thực hiện hoàn toàn ở nhà một cách thuận tiện. (H2)
15. Hãy tham gia cùng hàng triệu người đã thực hiện các bước để ngăn ngừa ung thư đại trực tràng. Sàng lọc là một trong những công cụ mạnh mẽ nhất mà chúng tôi có. Hãy cùng sử dụng và giữ gìn sức khỏe nhé. (J2)

| **Targeted Messages Phase 1** | | | | | | | | | | | | | | | | | | | |  |
| --- | --- | --- | --- | --- | --- | --- | --- | --- | --- | --- | --- | --- | --- | --- | --- | --- | --- | --- | --- | --- |
| Colorectal | Vietnamese | | F(1) | | H(3) | | E(5) | | A(7) | | B(9) | | | C(11) | | D(13) | | G(6) | |  |
| **Targeted Messages Phase 2** | | | | | | | | | | | | | | | | | | | | |
| Colorectal | Vietnamese | G(2) | | A(4) | | F(6) | | B(8) | | C(10) | | D(12) | E(1) | | H(14) | | I(3) | | J(15) | |

**Cervical cancer screening**

1. Vì ung thư cổ tử cung thường không có dấu hiệu trong giai đoạn đầu nên việc sàng lọc là rất quan trọng để phát hiện sớm khi mà việc điều trị có hiệu quả nhất. (D1)
2. Không có triệu chứng? Bạn vẫn cần xét nghiệm Pap. Ung thư cổ tử cung có thể diễn ra âm thầm nhưng gây chết người. Sàng lọc sớm là cách bảo vệ tốt nhất cho bạn. (F2)
3. Xét nghiệm Pap giúp tìm ra bất kỳ tế bào “tiền ung thư” nào, do đó nó có thể phát hiện ung thư cổ tử cung trước khi nó bắt đầu. (A1)
4. Xét nghiệm Pap đơn giản có thể cứu sống bạn bằng cách phát hiện sớm ung thư cổ tử cung. Đừng chờ đợi các triệu chứng xuất hiện. Hãy lên lịch để thực hiện sàng lọc sớm ngày hôm nay. (A2)
5. Bằng cách biến xét nghiệm Pap trở thành một phần trong thói quen chăm sóc sức khỏe của bạn, bạn đang thực hiện các bước chủ động để bảo vệ chống lại ung thư cổ tử cung. (B1)
6. Phát hiện sớm là chìa khóa để đánh bại ung thư cổ tử cung. Xét nghiệm Pap thường xuyên có thể phát hiện được nó trước khi nó bắt đầu. Hãy chăm sóc sức khỏe của bạn với một cuộc kiểm tra nhanh chóng. (E1/B2)
7. Việc tiếp cận xét nghiệm Pap đóng một vai trò quan trọng trong việc giảm tỷ lệ tử vong do ung thư cổ tử cung. (C1)
8. Xét nghiệm Pap nhanh chóng, dễ dàng và có thể là cứu cánh. Hãy tham gia cùng hàng triệu phụ nữ được khám sàng lọc thường xuyên và bảo vệ bản thân khỏi bệnh ung thư cổ tử cung. (C2)
9. Ung thư cổ tử cung không phải là một mối đe dọa. Xét nghiệm Pap thường xuyên có thể phát hiện sớm các vấn đề khi chúng có thể điều trị được tốt nhất. Hãy biến việc sàng lọc trở thành một phần thói quen sức khỏe của bạn. (D2)
10. Bạn có biết không? Tỷ lệ tử vong do ung thư cổ tử cung đã giảm đáng kể nhờ xét nghiệm Pap. Hãy là một phần của câu chuyện thành công này.Hãy tham gia sàng lọc. (F1/E2)
11. Hãy bảo vệ tương lai của bạn bằng xét nghiệm Pap. Đây là một trong những cách hiệu quả nhất để ngăn ngừa ung thư cổ tử cung. Hãy tăng cường sức khỏe cho chính bạn bằng các buổi sàng lọc thường xuyên. (G1/I2)
12. Chỉ mất vài phút để xét nghiệm Pap mỗi vài năm có thể tạo nên sự khác biệt trong suốt cuộc đời. Ưu tiên sức khỏe của bạn. Hãy lên lịch kiểm tra cho bạn. (G2)
13. Hãy là một anh hùng sức khỏe. Xét nghiệm Pap thường xuyên có thể phát hiện sớm ung thư cổ tử cung, giúp việc điều trị dễ dàng và hiệu quả hơn. Hãy đặt lịch hẹn khám sàng lọc ngay bây giờ. (H1/J2)
14. Xét nghiệm Pap không chỉ có tác dụng sàng lọc ung thư; nó mang lại sự yên tâm. Biết rằng bạn đang thực hiện các bước chủ động vì sức khỏe của mình là điều vô giá. (H2)

| **Targeted Messages Phase 1** | | | | | | | | | | | | | | | | | | | |  |
| --- | --- | --- | --- | --- | --- | --- | --- | --- | --- | --- | --- | --- | --- | --- | --- | --- | --- | --- | --- | --- |
| Pap | Vietnamese | | D(1) | | A(3) | | B(5) | | C(7) | | E(6) | | | F(10) | | G(11) | | H(13) | |  |
| **Targeted Messages Phase 2** | | | | | | | | | | | | | | | | | | | | |
| Pap | Vietnamese | F(2) | | A(4) | | B(6) | | C(8) | | D(9) | | E(10) | G(12) | | H(14) | | I(11) | | J(13) | |

General notes:

· Vietnamese CAB had preferences for first, second, and sometimes third messages but no preference after that- listed in the order presented beyond recommended messages

**ARABIC**

**Stage 1:** **Generic messages about missing preventive behaviors delivered to all participants**

عزيزي [weave_participant_arm_1] [name],

هذه الرسالة من مستشفى سان يسيدرو. تُظهر سجلاتنا أنك تفتقد واحدة أو أكثر من الخدمات الصحية الضرورية.

لتحديد موعد، يرجى الاتصال على الرقم (619)-6624100

Second message: واحدة أو أكثر. إن الاهتمام بصحتك أمر مهم لتقليل خطر الإصابة بالمرض والإعاقة والوفاة. برجاء الاتصال أو إرسال رسالة نصية على الرقم (619) 662-4100 لتحديد موعدك اليوم

Third message:
 تظهر سجلاتنا أنك لا تزال في عداد المفقودين التطعيم. إن الحصول على التطعيم يمكن أن يحميك أنت وأحبائك من الإصابة بالمرض. اتصل أو أرسل رسالة نصية على الرقم 619-662-4100 لتحديد موعدك اليوم.

Fourth message:

تظهر سجلاتنا أنك لا تزال تفتقد خدمة الرعاية الوقائية. يمكن أن يساعد الحصول على فحوصات الرعاية الوقائية في اكتشاف المشكلات في مرحلة مبكرة. اتصل أو أرسل رسالة نصية على الرقم 619-662-4100 لتحديد موعدك

**Stage 2: Messages selected for each participant depending on which vaccines and preventive care behaviors are missing**

**COVID-19 vaccine/** **لقاح كوفيد- ۱۹**

1. إذا أصبت بفيروس كورونا، فقد يعرض ذلك الأطفال وكبار السن في مجتمعك للخطر. (A1)
2. الأشخاص الذين تم تطعيمهم هم أقل عرضة لنشر فيروس كورونا إلى العائلة والأصدقاء. احصل على فرصتك وكن حاميًا، وليس موزعًا.(F1/C2)
3. لقاحات كوفيد مجانية ويسهل الحصول عليها. لا تأمين؟ لا مشكلة. دعنا نقوم بتطعيمك ونواصل المضي قدمًا.(H1)
4. اللقاح يحميك من متحورات كوفيد، تابع جرعاتك. اللقاحات مجانية بدون تامين . دعنا نساعدك للبقاء امنا(G1/B2)
5. حتى إذا كنت شاباً وبصحة جيدة، يمكن أن يفاجئك كوفيد بمشاكل خطيرة. اللقاح هو أفضل رهان لك للبقاء بصحة جيدة (D2)
6. إن اختيار الحصول على لقاح فيروس كورونا يمكن أن يحميك أنت وأحبائك من الإصابة بالمرض.(B1)
7. تذكر، كوفيد ليس مجرد نزلة برد. يمكن أن يكون خطيرًا. ولكن اللقاح يمكن أن يساعد في منع أسوأ النتائج. احمِ نفسك بلقاحك.(E1/F2)
8. عندما تحصل على التطعيم، فإن ذلك يساعد على تقليل خطر التعرض للآثار الضارة لفيروس كورونا.(C1)
9. (A2)كل لقاح يقربنا خطوة نحو إنهاء هذا الوباء. قم بدورك، احصل على التطعيم، ولننتقل معاً نحو الأفضل.
10. (I2)ملايين من الأشخاص قد تلقوا بأمان لقاح كوفيد. انضم إليهم، ولنعود إلى الأشياء التي نحبها بأمان وبسرعة.
11. يساعد كل شخص يتم تطعيمه على تقليل احتمالية إصابة أحبائه بالمرض.(D1)
12. لقاح كوفيد مثل درع لجسمك، يساعدك في محاربة الفيروس إذا تعرضت له. قَوِي درعك اليوم! (E2)
13. هل تعلم؟ اللقاح المضاد لكوفيد يقلل بشكل كبير من خطر وصولك إلى المستشفى. لنبقيك بصحة جيدة وآمنة. (G2)
14. تلقي لقاح كوفيد يساعد في الحفاظ على فتح مدارسنا ومكاتب عملنا. احمي مجتمعك وحافظ على استمرار الحياة. (H2)

| **Targeted Messages A** | | | | | | | | | | | | | | | | | | |  |
| --- | --- | --- | --- | --- | --- | --- | --- | --- | --- | --- | --- | --- | --- | --- | --- | --- | --- | --- | --- |
| COVID-19 | Arabic | | A(1) | | H(3) | | F(2) | | B(6) | | C(8) | | E(7) | | D(11) | | G(4) | |  |
| **Targeted Messages B** | | | | | | | | | | | | | | | | | | | |
| COVID | Arabic | C(2) | | B(4) | | D(5) | | F(7) | | A(9) | | I(10) | E(12) | G(13) | | H(14) | |  | |

· Arabic CAB thought warnings about disease and specifying symptoms would be most effective

**Flu vaccine**

1. أي شخص، حتى لو كان بصحة جيدة، يمكن أن يواجه مخاطر خطيرة مثل الالتهاب الرئوي بسبب الإنفلونزا.(B1)
2. الأطفال وكبار السن هم أكثر عرضة للإصابة بالإنفلونزا. قم بتطعيم نفسك للحفاظ على سلامة أحبائك. إنها خطوة .(B2)بسيطة بتأثير كبير
3. عندما تحصل على لقاح الأنفلونزا، فإنك لا تحمي نفسك فحسب، بل تحمي عائلتك أيضًا. (C1)

لقاحات الإنفلونزا تنقذ الأرواح. تظهر الدراسات أن لقاحات الإنفلونزا تقلل بشكل كبير من خطر الوفاة المرتبطة .

1. (F1/ .لدى الأطفال والبالغين
2. لقاحات الإنفلونزا هي أفضل دفاع لك ضد التغيب عن العمل وأيام الدراسة بسبب أمراض الإنفلونزا. حافظ على إنتاجيتك بالحصول على لقاح الإنفلونزا الآن.(E1/I2)
3. !حمِ صحتك في موسم الإنفلونزا هذا! يقلل لقاح الإنفلونزا من خطر الإقامة في المستشفى بسبب الإنفلونزا بنسبة تقريبية 40% بين البالغين. احصل على التطعيم اليوم!(A2)
4. هل تشعر بأنك بصحة جيدة؟ لا يزال عليك الحصول على لقاح الإنفلونزا. يمكن للأفراد الأصحاء نقل فيروس الإنفلونزا دون ظهور أعراض، مما يعرض صحة الآخرين للخطر. كن بطلا للمجتمع.(G1/G2)

من خلال الحصول على لقاح الأنفلونزا، يمكنك تقليل فرص اضطرارك لزيارة الطبيب بنسبة ٤۰-٥۰% (A1)

1. هل تشعر بالقلق بشأن فعالية لقاح الإنفلونزا؟ على الرغم من تفاوتها، التطعيم لا يزال الطريقة الأكثر فعالية للوقاية
2. من الإنفلونزا ومضاعفاتها. لا تنتظر، احصل على التطعيم الآن!(D2)
3. في كل عام، يمنع لقاح الأنفلونزا ملايين الأمراض وزيارات الأطباء المرتبطة بالأنفلونزا. انضم إلى هذه الأرقام واحصل على لقاح الأنفلونزا اليوم. صحتك تهمك.ا.(H1/H2)

يصيب موسم الإنفلونزا بشكل قوي، لكن الحصول على لقاح الإنفلونزا سهل وغالباً مجاني أو بتكلفة

1. منخفضة مع التأمين الصحي. تواصل معنا لكيفية الحصول على التطعيم بدون عناء.(F2)
2. (D1)يمكن أن يؤدي تخطي لقاح الإنفلونزا إلى تعريض الأشخاص الضعفاء لمخاطر جسيمة خلال موسم الإنفلونزا.
3. تذكر، لقاح الإنفلونزا لا يمكن أن يسبب لك الإنفلونزا. إنه وسيلة آمنة لحماية نفسك ومجتمعك من تهديد صحي خطير. انضم إلى مكافحة الإنفلونزا. (E2)
4. هل تعلم؟ الحصول على لقاح الإنفلونزا سنويًا هو أفضل طريقة للوقاية من فيروسات الإنفلونزا المتداولة كل عام. حافظ على تحديث تطعيم الإنفلونزا الخاص بك.(J2)

| **Targeted Messages A** | | | | | | | | | | | | | | | | | | | |  |
| --- | --- | --- | --- | --- | --- | --- | --- | --- | --- | --- | --- | --- | --- | --- | --- | --- | --- | --- | --- | --- |
| Flu | Arabic | | B(1) | | C(3) | | E(5) | | F(4) | | A(8) | | | H(10) | | G(7) | | D(12) | |  |
| **Targeted Messages B** | | | | | | | | | | | | | | | | | | | | |
| Flu | Arabic | B(2) | | C(4) | | A(6) | | G(7) | | D(9) | | F(11) | I(5) | | E(13) | | H(10) | | J(14) | |

**Breast Cancer screening**

1. إن إجراء تصوير الثدي بالأشعة السينية بشكل منتظم يمكن أن يقلل من فرص فقدان حياتك بسبب سرطان الثدي. (B1)
2. هل كنت تعلم؟ النساء اللاتي يحصلن على تصوير الثدي بالأشعة السينية بانتظام أقل عرضة للحاجة إلى علاجات عدوانية مثل الجراحة أو العلاج الكيميائي. اجعل الأمر بسيطًا، قم بالفحص. (E1/D2)

1. الكشف المبكر من خلال فحص الماموغرام يمكن أن ينقذ حياتك إذا تم اكتشاف سرطان الثدي (C1)
2. هل تحب حياتك؟ احصل على فحص الثدي. إنه خطوة صغيرة لقضية كبيرة: صحتك. الكشف المبكر يعني علاجًا أسهل.(C2)
3. فحوصات الماموغرام هي الخيار الأفضل لاكتشاف سرطان الثدي مبكرًا، مما يجعل العلاج أكثر سهولة. (D1)
4. ينقذ فحص الثدي الأرواح من خلال اكتشاف سرطان الثدي في مراحله المبكرة عندما يكون قابلاً للعلاج بشكل أكبر.

انضم إلى الملايين من النساء اللواتي يجرين الفحص كل عام. (H2)

1. عائلتك تحتاجك. يمكن لفحص الماموغرام السريع أن يساعد في ضمان بقائك معهم بصحة وقوة. احجزي موعد فحصك اليوم. (G1/G2)
2. سرطان الثدي لا يأتي دائمًا مع علامات تحذيرية. لذلك فإن فحوصات الثدي أمر بالغ الأهمية. فهي ترى ما لا نستطيع رؤيته، وتكتشف السرطان في مراحله المبكرة. (F2)
3. مخاطر الإصابة بسرطان الثدي تختلف من امرأة لأخرى. ولكن كل امرأة تستحق أفضل فرصة للتغلب عليه. فحوصات الماموغرام هي تلك الفرصة. (H1/I2)
4. هل تعتقدين أنك مشغولة للغاية لإجراء تصوير الثدي بالأشعة السينية؟ يستغرق الأمر وقتًا أقل من استراحة تناول القهوة ويمكن أن يمنحك المزيد من أعياد الميلاد للاحتفال بها. (F1/E2)
5. الطمأنينة لا تقدر بثمن. توفر فحوصات الثدي ذلك من خلال التأكد من أنك تتخذ خطوات استباقية ضد سرطان الثدي. قم بتحديد موعد لك. (J2)
6. تصوير الثدي بالأشعة السينية سريع، حوالي ۲۰دقيقة فقط، ويمكن أن يساعد في ضمان قضاء وقت ممتع أكثر مع أحبائك. (A1)
7. لا تنتظر ظهور الأعراض. يمكن لفحوصات الثدي اكتشاف سرطان الثدي في مراحل مبكرة، عندما يكون قابلاً للعلاج بشكل أفضل. قم بتحديد موعدك اليوم. (A2)
8. الفحص الدوري لسرطان الثدي هو عن الحماية. فحص الثدي الذي يستغرق 20 دقيقة الآن يمكن أن يحدث فارقًا كبيرًا في المستقبل. دعونا نقوم بالفحص الآن.(B2)

| **Targeted Messages A** | | | | | | | | | | | | | | | | | | | |  |
| --- | --- | --- | --- | --- | --- | --- | --- | --- | --- | --- | --- | --- | --- | --- | --- | --- | --- | --- | --- | --- |
| Mammogram | Arabic | | B(1) | | C(3) | | D(5) | | G(7) | | H(9) | | | E(2) | | F(10) | | A(12) | |  |
| **Targeted Messages B** | | | | | | | | | | | | | | | | | | | | |
| Mammogram | Arabic | D(2) | | C(4) | | H(6) | | F(8) | | G(7) | | I(9) | J(11) | | E(10) | | A(13) | | B(14) | |

**Colorectal cancer screening**

1. هناك طرق آمنة مختلفة لإجراء فحص سرطان القولون والمستقيم، بما في ذلك الاختبارات التي يمكنك إجراؤها في المنزل. (A1)
2. فحص سرطان القولون والمستقيم يمكن أن ينقذ حياتك. إنه بسيط وهناك عدة خيارات، بما في ذلك اختبارات سهلة يمكنك القيام بها في المنزل. لا تنتظر، احصل على الفحص! (A2)
3. يعد سرطان القولون والمستقيم ثالث أكثر أنواع السرطانات القاتلة شيوعًا، خاصة مع التقدم في السن. (B1)
4. قد تشعر بأنك بصحة جيدة ولكن قد تكون مصابًا بسرطان القولون والمستقيم. يساعد الفحص في اكتشافه في مراحله المبكرة، حتى بدون ظهور أي أعراض. إنه خطوة يمكنك اتخاذها لطمأنينة البال. (C2)
5. تعد اختبارات الكشف عن سرطان القولون والمستقيم أمرًا بالغ الأهمية لأنه يمكن اكتشافه مبكرًا، عندما يكون العلاج أفضل. (C1)
6. الفحص ينقذ الأرواح. الأشخاص الذين يتم فحصهم بانتظام بحثًا عن سرطان القولون والمستقيم لديهم معدل بقاء على قيد الحياة بنسبة ۹۰٪ عندما يتم اكتشاف السرطان مبكرًا. (H1/I2)
7. لا تظهر علامات سرطان القولون والمستقيم دائمًا، ولهذا السبب يعد الفحص أمرًا حيويًا للكشف عنه قبل أن يصبح مشكلة. (D1)
8. أكثر من ٤٥؟ حان الوقت لإجراء فحص سرطان القولون والمستقيم. يزيد العمر من المخاطر، والفحص هو أفضل وسيلة لحماية نفسك. (F1/E2)
9. منع ما يمكن الوقاية منه. غالبًا ما يمكن الوقاية من سرطان القولون والمستقيم من خلال الفحص عن طريق العثور على الخلايا وإزالتها قبل أن تتحول إلى سرطان. (E1)
10. هل تشعر بالقلق من التوتر؟ خيارات الفحص الحديثة أكثر راحة من أي وقت مضى. يمكن إجراء بعضها تمامًا في المنزل، وفقًا لراحتك. (H2)
11. من الواجب منع ماهو القابل للمنع. يمكن في كثير من الأحيان منع سرطان القولون والمستقيم من خلال الفحص الطبي الوقائي بالعثور على الأورام الحميدة وإزالتها قبل تحولها إلى سرطان. (D2)

1. تأجيل فحص سرطان القولون والمستقيم؟ معظم الأشخاص الذين يخضعون للفحص يندمون على شيء واحد فقط: عدم القيام بذلك عاجلاً. السيطرة على صحتك. (G1/F2)

1. الفحص الطبي الوقائي لسرطان القولون والمستقيم أكثر من مجرد اختبار - إنه وسيلة لتولي السيطرة على صحتك. العديد من خطط التأمين تغطي تكلفته. (G2)
2. *هل تعلم؟ يمكن للفحص الطبي الوقائي اكتشاف سرطان القولون والمستقيم في مراحله المبكرة عندما يكون قابلًا للعلاج بشكل أكبر. الكشف المبكر يحدث فارقاً كبيراً. قم بجدولة فحصك اليوم. (B2)*

1. انضم إلى الملايين الذين اتخذوا خطوات لمنع سرطان القولون والمستقيم. الفحص الطبي الوقائي هو أحد أقوى الأدوات التي لدينا. لنستخدمها ونبقى أصحاء معًا. (J2)

| **Targeted Messages A** | | | | | | | | | | | | | | | | | | | |  |
| --- | --- | --- | --- | --- | --- | --- | --- | --- | --- | --- | --- | --- | --- | --- | --- | --- | --- | --- | --- | --- |
| Colorectal | Arabic | | A(1) | | B(3) | | C(5) | | D(7) | | E(9) | | | F(8) | | G(12) | | H(6) | |  |
| **Targeted Messages B** | | | | | | | | | | | | | | | | | | | | |
| Colorectal | Arabic | A(2) | | C(4) | | I(6) | | E(8) | | H(10) | | D(11) | G(13) | | F(12) | | B(14) | | J(15) | |

**Cervical cancer screening**

1. نظرًا لأن سرطان عنق الرحم لا يظهر في كثير من الأحيان أي علامات في مراحله الأولية، فإن الفحص ضروري لاكتشافه مبكرًا عندما يكون العلاج أكثر فعالية. (D1)
2. هل كنت تعلم؟ انخفضت الوفيات الناجمة عن سرطان عنق الرحم بشكل ملحوظ بفضل اختبارات مسحة عنق الرحم. كن جزءا من قصة النجاح هذه. قم بالفحص. (F1/E2)
3. يلعب الوصول إلى اختبارات عنق الرحم دورًا حيويًا في الحد من الوفيات الناجمة عن سرطان عنق الرحم. (C1)
4. بضع دقائق فقط لإجراء فحص عنق الرحم كل بضع سنوات يمكن أن تحدث فارقًا في حياتك. اعتبر صحتك أولوية. قم بحجز فحصك. (G2)
5. الاكتشاف المبكر هو مفتاح التغلب على سرطان عنق الرحم. يمكن لاختبارات مسحة عنق الرحم المنتظمة اكتشافه قبل أن يبدأ. تولى مسؤولية صحتك من خلال إجراء فحص سريع. (E1/B2)
6. يقوم فحص عنق الرحم بأكثر من فحص للسرطان؛ بل يوفر الطمأنينة. معرفة أنك تتخذ خطوات استباقية لصحتك لا تقدر بثمن. (H2)
7. قم بحماية مستقبلك من خلال اختبار مسحة عنق الرحم. إنها واحدة من أكثر الطرق فعالية للوقاية من سرطان عنق الرحم. تمكين نفسك مع عروض منتظمة. (G1/I2)
8. كن بطلاً في مجال الصحة. يمكن لاختبارات مسحة عنق الرحم المنتظمة اكتشاف سرطان عنق الرحم مبكرًا، مما يجعل العلاج أسهل وأكثر فعالية. احجز موعد الفحص الخاص بك الآن. (H1/J2)
9. الفحوصات العنقية سريعة وسهلة وقد تكون محافظة على الحياة. انضمي إلى الملايين من النساء اللواتي يجرين الفحص بانتظام وحمي نفسك من سرطان عنق الرحم. (C2)
10. يساعد اختبار مسحة عنق الرحم في العثور على أي خلايا "محتملة للتسرطن"، حتى يتمكن من اكتشاف سرطان عنق الرحم قبل أن يبدأ. (A1)
11. من خلال جعل اختبار مسحة عنق الرحم جزءًا من روتين الرعاية الصحية الخاص بك، فإنك تتخذ خطوات استباقية للحماية من سرطان عنق الرحم. (B1)
12. سرطان عنق الرحم لا يجب أن يكون تهديدًا. يمكن للفحوص العنقية الدورية اكتشاف المشاكل في مراحلها المبكرة عندما يكون العلاج أكثر فعالية. اجعل الفحص جزءًا من روتينك الصحي. (D2)
13. اختبار عنق الرحم البسيط قد ينقذ حياتك من خلال اكتشاف سرطان عنق الرحم في مراحله المبكرة. لا تنتظر ظهور الأعراض. قم بجدولة فحصك اليوم. (A2)
14. لا توجد أعراض؟ لا يزال عليك إجراء فحص باب الرحم. يمكن أن يكون سرطان عنق الرحم صامتًا ولكنه قاتل. الكشف المبكر هو أفضل دفاع لديك. (F2)

| **Targeted Messages A** | | | | | | | | | | | | | | | | | | | |  |
| --- | --- | --- | --- | --- | --- | --- | --- | --- | --- | --- | --- | --- | --- | --- | --- | --- | --- | --- | --- | --- |
| Pap | Arabic | | D(1) | | C(3) | | E(5) | | G(7) | | H(8) | | | A(10) | | B(11) | | F(2) | |  |
| **Targeted Messages B** | | | | | | | | | | | | | | | | | | | | |
| Pap | Arabic | E(2) | | G(4) | | H(6) | | B(5) | | C(9) | | J(8) | D(12) | | A(13) | | I(7) | | F(14) | |
